# Host–Pathogen Coevolution: The Selective Advantage of *Bacillus thuringiensis* Virulence and Its Cry Toxin Genes

**DOI:** 10.1371/journal.pbio.1002169

**Published:** 2015-06-04

**Authors:** Leila Masri, Antoine Branca, Anna E. Sheppard, Andrei Papkou, David Laehnemann, Patrick S. Guenther, Swantje Prahl, Manja Saebelfeld, Jacqueline Hollensteiner, Heiko Liesegang, Elzbieta Brzuszkiewicz, Rolf Daniel, Nicolaas K. Michiels, Rebecca D. Schulte, Joachim Kurtz, Philip Rosenstiel, Arndt Telschow, Erich Bornberg-Bauer, Hinrich Schulenburg

**Affiliations:** 1 Department of Evolutionary Ecology and Genetics, Zoological Institute, Christian-Albrechts-University of Kiel, Kiel, Germany; 2 Department of Animal Evolutionary Ecology, Institute of Evolution and Ecology, University of Tuebingen, Tuebingen, Germany; 3 Institute for Evolution and Biodiversity, University of Muenster, Muenster, Germany; 4 Goettingen Genomics Laboratory, Institute of Microbiology and Genetics, Georg-August-University of Goettingen, Goettingen, Germany; 5 Department of Behavioural Biology, University of Osnabrueck, Osnabrueck, Germany; 6 Institute for Clinical Molecular Biology, Christian-Albrechts-University, Kiel, Germany; Stanford University, UNITED STATES

## Abstract

Reciprocal coevolution between host and pathogen is widely seen as a major driver of evolution and biological innovation. Yet, to date, the underlying genetic mechanisms and associated trait functions that are unique to rapid coevolutionary change are generally unknown. We here combined experimental evolution of the bacterial biocontrol agent *Bacillus thuringiensis* and its nematode host *Caenorhabditis elegans* with large-scale phenotyping, whole genome analysis, and functional genetics to demonstrate the selective benefit of pathogen virulence and the underlying toxin genes during the adaptation process. We show that: (i) high virulence was specifically favoured during pathogen–host coevolution rather than pathogen one-sided adaptation to a nonchanging host or to an environment without host; (ii) the pathogen genotype BT-679 with known nematocidal toxin genes and high virulence specifically swept to fixation in all of the independent replicate populations under coevolution but only some under one-sided adaptation; (iii) high virulence in the BT-679-dominated populations correlated with elevated copy numbers of the plasmid containing the nematocidal toxin genes; (iv) loss of virulence in a toxin-plasmid lacking BT-679 isolate was reconstituted by genetic reintroduction or external addition of the toxins. We conclude that sustained coevolution is distinct from unidirectional selection in shaping the pathogen's genome and life history characteristics. To our knowledge, this study is the first to characterize the pathogen genes involved in coevolutionary adaptation in an animal host–pathogen interaction system.

## Abstract 2

A combination of experimental evolution with large-scale phenotyping, genomics and functional genetics reveals the specific role of virulence and toxin genes during the evolutionary adaptation of a pathogen to an animal host.

## Author Summary

Evolution can be extremely fast and dramatic, especially when infectious disease agents such as bacterial pathogens engage in a continuous arms race with their host organism. Rounds of novel pathogen attack strategies and associated host counterdefenses conspire to drive host–pathogen coevolution and biological innovation. To better understand the underlying genetic mechanisms and the exact trait characteristics under selection, we conducted experimental evolution using a simple host–pathogen model system (nematode versus bacterium) under controlled laboratory conditions. We analysed the associated adaptive changes in real time using large-scale phenotyping, population whole genome sequencing, and genetic analysis of the identified candidate genes. We show that coevolution (rather than one-sided adaptation) particularly favors and maintains pathogen virulence, and that two specific toxin genes significantly influence this virulence during coevolution.

## Introduction

Antagonisms are often at the heart of rapid evolutionary change. One prime example for such antagonism is given by the interaction between host and pathogen. By definition, pathogens have a negative effect on host fitness, favouring selection of enhanced defence mechanisms in the affected hosts. If pathogen fitness depends on the host, then host defence can be detrimental for the pathogen, leading to selection for novel attack mechanisms. When the interaction persists over time, the ongoing cycles of adaptation and counteradaptation can produce one of the highest selective pressures known in nature [1–4]. There are numerous examples of the resulting rapid evolutionary responses during host–pathogen coevolution, including taxonomically diverse host systems such as bacteria [5,6], plants [7–9], invertebrates [10–13], and vertebrate animals [14]. In spite of its potential importance as a major driver of evolution, two core features of the coevolutionary dynamics are as yet only poorly understood [3,15]: (i) which trait functions are specifically under selection during coevolution when antagonists reciprocally coadapt to each other, rather than only one adapting while the other remains unchanged? (ii) Which genes and genetic mechanisms underlie adaptation during coevolution, particularly when rapid changes are required to keep up with the coevolving antagonist?

To date, very few studies have evaluated the selective consequences of coevolution relative to one-sided adaptation, and these have mainly used bacteria–phage interaction models [16–19]. For instance, the *Pseudomonas fluorescens*-infecting phage Φ2 was evolved in the presence of either its coevolving host or a nonevolving host, leading to the unique emergence and persistence of different phage infection varieties under the coevolution conditions [19]. Similarly, it is mainly bacteria–phage systems that have previously been used to dissect the genetics of host–pathogen coevolutionary change [16–18,20]. One of the recent examples was based on controlled coevolution of phage λ and its host *Escherichia coli*, resulting in a sequence of reciprocal adaptations in the phage to use different host receptors and in the host to alter the targeted receptors or prevent uptake of phage DNA [20]. Similar information for multicellular host interaction models is as yet scarce (e.g., [13]). Such information is essential for a more general understanding of the postulated impact of coevolution on the evolution of organisms [1,2,4] and, more precisely, it will help us understand which exact trait functions are under selection during sustained, reciprocally antagonistic interactions, how quickly changes can be achieved during evolution, and what the most likely resulting selection dynamics are [2,3,15].

**Fig 1:**
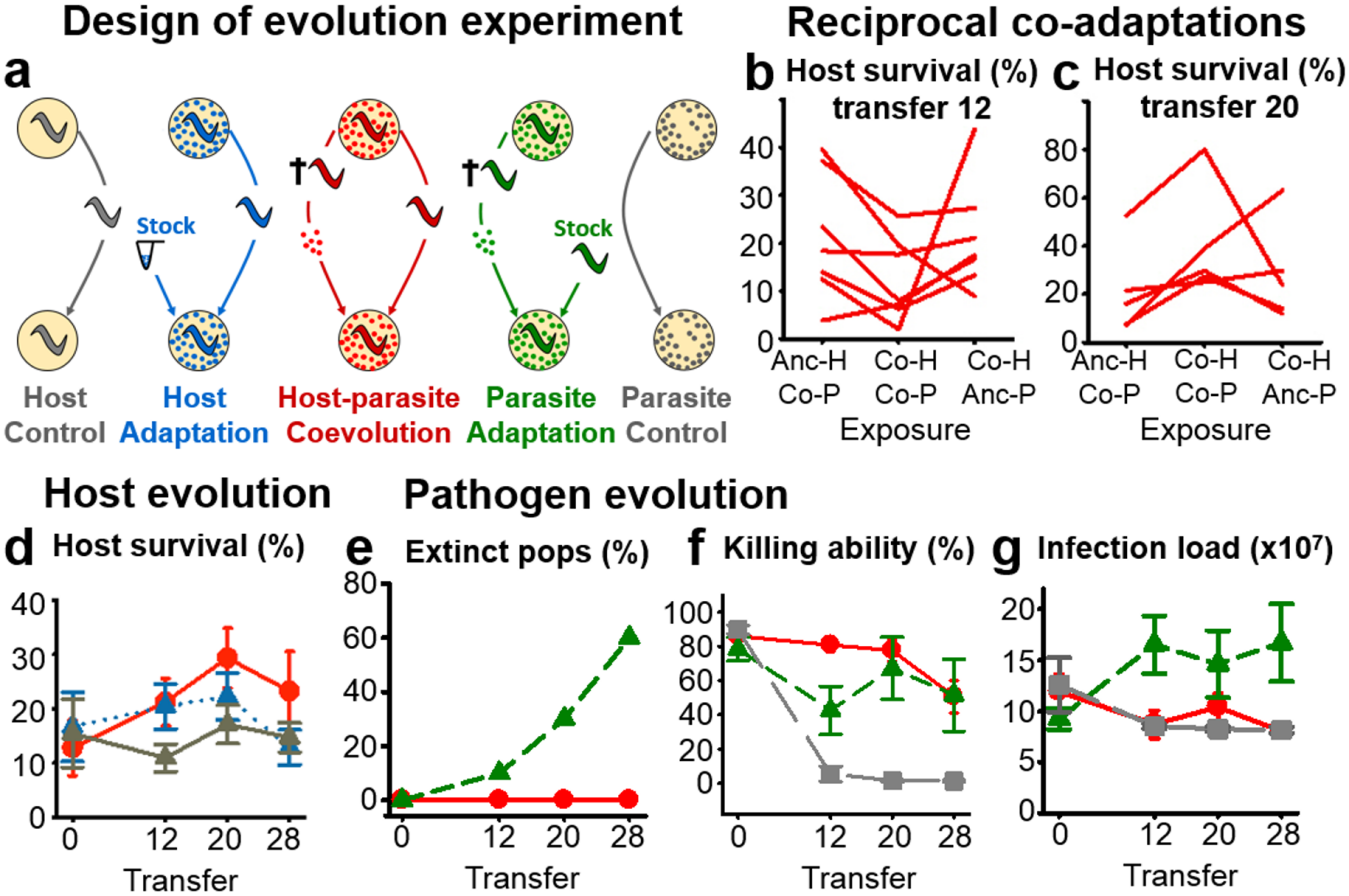
Experimental host–pathogen coevolution causes phenotypic changes in both antagonists. **A,** The five evolution treatments: (i) host control (grey) adapting to general laboratory conditions in the absence of the pathogen, (ii) host one-sided adaptation (blue) where the host adapted to the nonevolving, ancestral pathogen taken from a frozen stock culture at each transfer, (iii) host–pathogen coevolution (red) during which both antagonists were continuously forced to coevolve to each other, (iv) pathogen one-sided adaptation (green) where the pathogen adapted to the nonevolving, ancestral host population taken from a frozen stock culture at each transfer; and (v) pathogen control (grey) adapting to general laboratory conditions in the absence of the host. **B–C,** Analysis of reciprocal coadaptations in host survival and pathogen killing ability *(y*-axis) by comparing (along the *x*-axis) exposures of coevolved hosts with coevolved pathogens from the same replicate population and time point (indicated by Co-H Co-P in the middle of the panels) with either coevolved hosts from the same replicate exposed to ancestral pathogens (Co-H Anc-P, right side) or ancestral hosts exposed to coevolved pathogens from the same replicate (Anc-H Co-P, left side). Results are given for transfers 12 (**B**) and 20 (**C**) separately. The lines connect the results for particular replicate populations of the coevolution treatment. **D,** Survival of evolved host populations from different treatments (colors as in Fig 1A) upon exposure to the ancestral pathogen. **E,** Pathogen population extinctions under one-sided adaptation (green) and coevolution (red). **F–G**, Analysis of evolved pathogen populations from different treatments (colors as in Fig 1A) upon exposure to the ancestral host, including pathogen killing ability (measured as host death rate in %) (**F**) and pathogen infection load (**G**). Bars denote standard error. The original data is provided in S1 Data, and the results of the corresponding statistical analyses are given in S1 Table, S2 Table, S3 Table, S4 Table, and S5 Table.

Here, we addressed these questions by comparing alternative selection regimes in controlled evolution experiments, using an animal host interaction model, consisting of the nematode *Caenorhabditis elegans* as host and its natural pathogen *Bacillus thuringiensis* [21]. The Gram-positive bacterium *B*. *thuringiensis* is of economic importance as a pest control agent [22,23] and infects insect or nematode hosts upon oral uptake via toxin-mediated destruction of intestinal cells and expression of additional virulence factors [22,23]. The interaction between *B*. *thuringiensis* and *C*. *elegans* was previously established as an experimental evolution model for studying the consequences of coevolution [21,24–27]. We have now used this interaction model for a new experimental design that consisted of five distinct evolution treatments (Fig 1A; see Materials and Methods), namely: (i) host control, where the host adapted to general laboratory conditions in the absence of pathogenic *B*. *thuringiensis*; (ii) host one-sided adaptation, where the host adapted to the ancestral pathogenic *B*. *thuringiensis*, taken from a frozen stock at each transfer step; (iii) host–pathogen coevolution, where both host and pathogen coadapted to their continuously coevolving antagonist; (iv) pathogen one-sided adaptation, where the pathogen adapted to the ancestral *C*. *elegans* population, taken from a frozen stock at each transfer step; and (v) pathogen control, where the pathogen adapted to general laboratory conditions in the absence of a host. The evolution experiment was specifically designed to provide identical conditions for the different treatments, except for the presence of a coevolving antagonist, a nonevolving antagonist (i.e., the ancestral antagonist), or no antagonist. Thus, the design allowed us to assess the unique consequences of coevolutionary adaptation, as opposed to one-sided adaptation and laboratory adaptation. The *C*. *elegans*–*B*. *thuringiensis* system was chosen because it enables a high level of control over the evolutionary interaction and subsequent analyses. This is due to the fact that *C*. *elegans* and *B*. *thuringiensis* can be efficiently purified from each other during the transfer steps, preventing any unintended coevolution from occurring in the evolution treatments, and both can be cryopreserved. This cryopreservation allows identical cultures of the stock ancestral antagonists to be used for the respective one-sided adaptation treatment throughout the entire experiment and enables evolved populations to be frozen for later parallel phenotypic characterizations.

Based on this experimental setup, we compared the consequences of reciprocal coevolution with those resulting from related selective pressures. We here present our findings on the evolved phenotypic changes across time, combined with results from population whole genome sequence analysis and a subsequent functional genetic assessment.

## Results/Discussion

### Differences in Experimental Selection Conditions Lead to Multiple Distinct Changes in Host and Pathogen

After completion of the evolution experiment (Fig 1A), phenotypic changes were assessed for both host and pathogen. Evolved *B*. *thuringiensis* and *C*. *elegans* material, which was frozen at regular intervals during the evolution experiment (see Materials and Methods), was thawed and examined in parallel to assess phenotypic variation across time and evolution treatments. We considered a total of 86 evolved pathogen and 91 evolved host populations (covering three evolution treatments for each antagonist, with three transfer time points per treatment and up to ten independent replicate populations per treatment per time point), as well as the ancestral populations. For these, traits of relevance for the interaction were studied in the presence of either the coevolving antagonist from the same replicate population at a specific time point or the respective ancestral antagonist (see below). We characterized several proxies for host resistance sensu lato (survival, body size, population size, and infection load), proxies for pathogen virulence sensu lato (killing ability and pathogen effects on host body size and host population size), and pathogen infection load (see Materials and Methods). We additionally assessed the ability of the pathogen to form biofilms and the resulting competitive advantage on different nutritional agar media (Materials and Methods), as biofilm formation was found to be common in some of the evolution treatments. Our statistical analysis of the obtained data always included adjustment of significance levels through the false discovery rate (FDR) [28] to account for increased type I errors.

Our analysis revealed specific coadaptations between host and pathogen during the coevolution treatment. In particular, we compared the survival of coevolved hosts exposed to coevolved pathogens from the same time point and replicate to that of coevolved hosts from the same replicate, which were exposed to ancestral pathogens, or to that of coevolved pathogens from the same replicate, which were exposed to ancestral hosts. For this analysis, we included all replicates, for which data was available for all three types of exposures (seven for transfers 12 and 28; five for transfer 20). In the absence of reciprocal coadaptations, we would expect the coevolved–coevolved combinations to produce phenotypic values that are either identical to or the average of those from the coevolved–ancestral combinations. This is clearly not the case for transfers 12 and 20, for which the coevolved–coevolved combinations consistently produced either lower (for transfer 12) or higher (for transfer 20) survival than the corresponding exposures to the ancestral antagonists (Fig 1B and 1C). For these transfers, the comparisons are significantly different except for the comparison to the coevolved pathogens from transfer 20 exposed to the ancestral host, which still indicated a statistical trend (*p* < 0.1; S1 Table). At transfer 28, we could not identify any significant variation. We conclude that the experimental evolution conditions allowed the antagonists to reciprocally coadapt to each other within considerably short time periods of only 12 transfers.

We next assessed variation among evolution treatments for the host. Comparability of populations from different treatments was ensured by exposing all evolved hosts to the ancestral pathogens, followed by measurement of the various phenotypic traits. Our results showed that survival in the presence of the ancestral pathogen significantly increased during coevolution but not in other treatment conditions, suggesting evolution of increased host resistance sensu lato in the presence of a coevolving antagonist (Fig 1D, S2 Table, and S3 Table), generally consistent with above analysis of reciprocal coadaptations. None of the other considered traits showed significant variation (S2 Table, S3 Table).

We performed a similar comparison for the pathogen, based on the exposure of evolved pathogen populations from the various treatments to the ancestral host population. Intriguingly, pathogen one-sided adaptation caused extinction (i.e., no host killing, as pathogens were only transferred from dead hosts) in more than half of the pathogen populations. This was not observed under coevolution conditions (Fig 1E). The pathogen's ability to kill the host and reduce host body size and host population size (i.e., virulence sensu lato) was generally maintained during coevolution, while it was decreased transiently under pathogen one-sided adaptation and lost during pathogen control evolution (Fig 1F, S1 Fig, and S4 Table). The observed variation in killing ability was significant among all evolution treatments, while the pathogen's effect on host body size differed significantly between the control and each of the other treatments, and the pathogen's effect on host population size only showed a significant difference between the coevolution and control treatments (S5 Table). In contrast, pathogen infection load was significantly higher under one-sided adaptation than the other two treatments, which did not show significant variation from each other (Fig 1G, S4 Table, and S5 Table).

**Fig 2:**
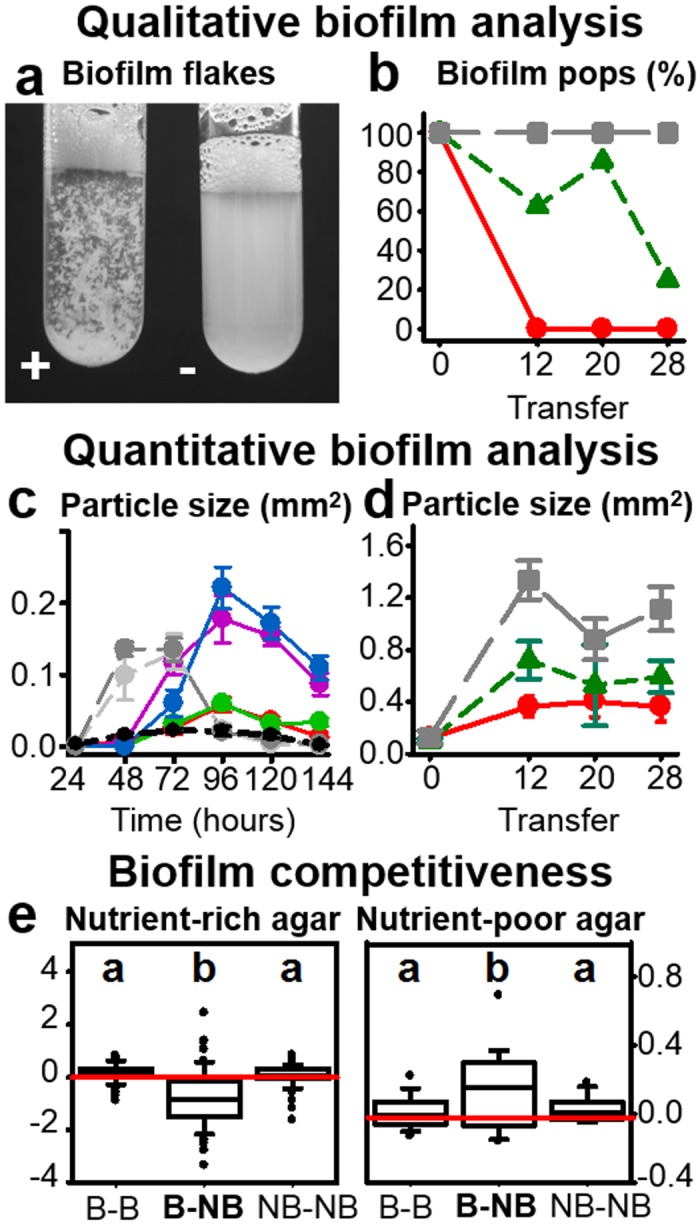
Variation of evolved *B*. *thuringiensis* in biofilm formation. **A**, Qualitative assessment of the presence (indicated by +) or absence (-) of biofilm flakes in evolved bacterial populations, washed off assay plates after 48 h of growth and inspected by eye in tubes. **B,** Ability of *B*. *thuringiensis* populations to form biofilms (% of populations) using the qualitative assay of Fig 2A. Red indicates coevolution, green one-sided adaptation, and grey control evolution. **C,** Quantification of the temporal dynamics of biofilm formation by measuring mean particle size during bacterial growth on plates across time; results for four evolved clones and three ancestral strains; grey shades indicate three of the ancestral strains (light grey: BT-247; dark grey: BT-246; black: BT-679), red a coevolved clone, green a one-sided adapted clone that is able to form biofilms, purple a one-sided adapted clone unable to form biofilms, and blue a non-biofilm-forming control-evolved clone. **D,** Similar quantification of mean biofilm particle size after growth on plates for 96 h for the evolved populations across transfers from the evolution experiment. Red indicates coevolution, green one-sided adaptation, and grey control evolution. **E**, Competitive ability of biofilm-forming (B) versus non-biofilm-forming (NB) clones on nutrient-rich nematode growth medium (left) or nutrient-poor peptone-free medium (right). Two clones always competed with each other in a paired setup. The value for the second listed phenotype was subtracted from the value for the first listed phenotype (e.g., B–NB, value for biofilm-producer minus value for non-biofilm-producer; see combinations on *x*-axis) to calculate a competitiveness index (*y*-axis). The red horizontal line indicates a value of zero (i.e., no difference). Different letters on top indicate significant variation between the different combinations. The original data is given in S2 Data and the corresponding statistical results in S6 Table and S7 Table.

We subsequently assessed the bacteria's ability to form biofilms. Biofilm formation is a dynamic process, including (i) accumulation of planktonic bacterial cells; (ii) maturation of the bacterial community and first differentiation of cell types; (iii) production of a robust extracellular matrix by specifically differentiated cells (i.e., biofilm production); (iv) disintegration of an aged biofilm; and (v) dispersal of planktonic cells that emerge from the biofilm [29]. Biofilm formation was characterized using independent qualitative and quantitative measurements (Materials and Methods). The qualitative analysis revealed that biofilm formation was significantly more common during control evolution, being lost under coevolution and, to a lesser extent, one-sided adaptation (Fig 2A and 2B, S2 Fig, and S6 Table). Two separate quantitative analyses of individual clones or evolved populations consistently revealed that biofilm particle size was larger for control-evolved and avirulent bacteria (Fig 2C and 2D). To further investigate the causes of treatment variation in biofilm formation, we tested the bacteria's competitive ability under either low nutrient conditions (as used during experimental evolution; Materials and Methods) or high nutrient conditions. We directly competed two bacterial clones in a paired setup, either (i) a biofilm-forming versus a non-biofilm-forming clone (test comparison), (ii) two biofilm-forming clones (first control), and (iii) two non-biofilm-forming clones (second control). We found that the competiveness of biofilm-producing clones was significantly higher on the nutrient-poor medium but significantly lower under nutrient-rich conditions (Fig 2E, S7 Table). Our results thus suggest that the low nutrient medium used during the main part of the evolution protocol (Materials and Methods) selectively favours the maintenance of biofilm-forming bacteria, which is of particular relevance in the absence of a host, as under the control conditions.

**Fig 3:**
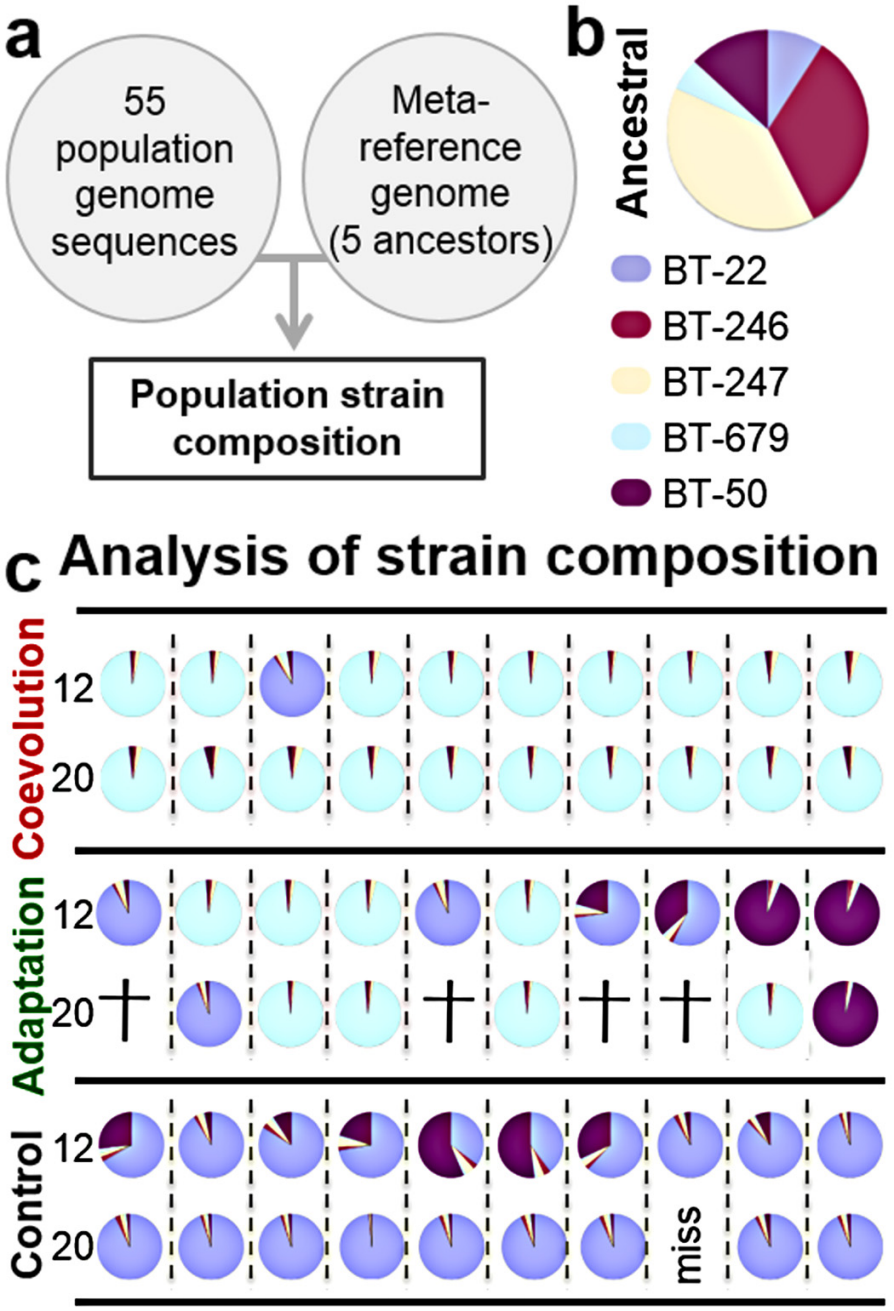
Broad-scale genomic analysis reveals clonal selection during experimental evolution. **A,** Genome analysis workflow: A metareference genome created from five genomes representative of the ancestral population was used for sequence read mapping and subsequent identification of strain composition for 55 evolved populations. **B–C,** Pie charts show pathogen strain composition of the ancestral and the evolved populations from ten replicates per treatment (horizontal axis) and two time points (transfer 12 and 20). Coloured slices indicate the relative abundance of the various *B*. *thuringiensis* strains. Crosses indicate extinction of replicates and "miss" that genetic material for the population was unavailable. The data is given in S3 Data.

**Fig 4:**
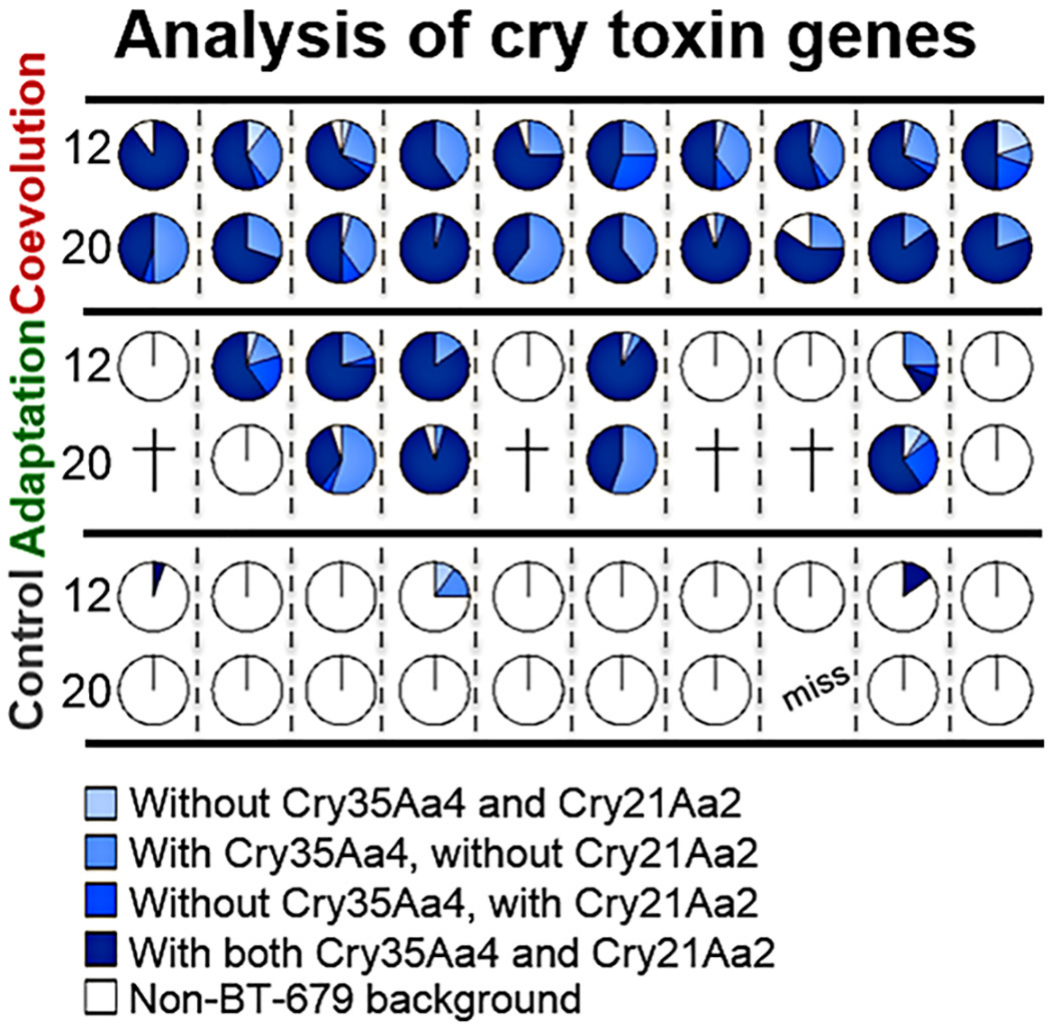
Frequency of BT-679 toxin genes *cry21Aa2* and *cry35Aa4* among the evolved replicate populations. The different shades of blue indicate alternative combinations of toxin genes present, as indicated. The toxin genes were all restricted to evolved clones of the BT-679 background (i.e., horizontal transfer was not detected). The top two rows refer to the coevolved, the middle two rows to one-sided adapted, and the bottom two rows to the control evolved replicate populations. Replicate populations are given along the horizontal axis. Data is shown for both transfer 12 and 20 and a total of 55 replicate populations. Crosses indicate extinction of replicates and "miss" that genetic material for the population was unavailable. The original data is shown in S4 Data.

Taken together, our phenotypic analysis reveals that the different selection conditions of the evolution experiment result in distinct phenotypic changes in both antagonists, especially for the pathogen. Our results thus extend the few reports, all based on bacteria–phage interaction models, which previously contrasted coevolution with one-sided adaptation for the pathogen and identified unique changes under coevolutionary adaptation [16–19]. Importantly, our new findings now suggest that the different selection conditions favour pathogen characteristics that are likely of relevance during different phases of its life cycle and that may also increase extinction risk in the absence of a coevolving host (i.e., in the one-sided adaptation treatment). At first, prior to host infection, *B*. *thuringiensis* must ensure persistence in an unfavourable environment [23], for instance through biofilm formation [29,30]. This stage of the life cycle was under specific selection during control evolution without a host, where the pathogen was maintained in a nutrient-poor environment (Fig 2). The ability to form biofilms coincided with a loss of pathogenicity (Figs 1F and 2B), possibly suggesting a life history trade-off. Thereafter, following host entry, *B*. *thuringiensis* is known to pass through two phases during the infection process [23]. The first phase is characterized by toxin-mediated tissue damage, which weakens the host, easing access to nutritional resources. As toxins ultimately cause host death [22,23], selection on this step may lead to variation in host killing ability. Thus, the toxin effects appear of particular selective benefit during coevolution (Fig 1B, 1C, and 1F), possibly because of ongoing resistance evolution in the host (Fig 1B–1D). The second phase starts when the host is weakened or already dead, and when pathogens increase replication rate [23], leading to elevated infection load. During this latter phase, bacteria may have an advantage if they do not pay the cost of toxin production and/or replication of the toxin-containing plasmid and can thus grow faster than toxin-producing and/or plasmid-bearing cells [23,31]. This step appears to be under particular selection in the one-sided adaptation treatment, for which a significantly higher infection load was recorded (Fig 1G). Moreover, the duration of this second phase in the pathogen one-sided adaptation treatment (as determined by the characteristics of the ancestral host population used) seems to represent a "turning point" where chance can favour either toxin-bearing or toxin-lacking bacteria, as demonstrated below by the results of the genomic analysis (see below and Figs 3C and 4) and indicated also by the overall decreased virulence for this treatment (Fig 1F). If an avirulent genotype spreads to fixation in one of these populations, the bacteria would no longer be able to infect new hosts because of the absence of toxins, thus potentially explaining the high extinction rate under these conditions (Fig 1F; see below for further details).

### Differences in Experimental Selection Conditions Favour Distinct Pathogen Genotypes

Genetic changes in the pathogen were explored through whole genome sequence analysis and a toxin gene screen of *B*. *thuringiensis* populations from three time points (transfers 0, 12, and 20). As a basis for our analysis, we first assembled reference genomes for five strains present in the ancestral population and established a novel analysis pipeline that ensured reliable variant detection in genetically variable populations (Fig 3A, S3 Fig, S4 Fig, S8 Table, S9 Table, Materials and Methods). This pipeline was used to analyse a total of 56 whole population genomes (three evolution treatments and two time points, with up to ten replicate populations each, plus the ancestral population). Based on the analysis, we identified a dramatic change in strain composition from the ancestral to the evolved populations. While several strains were abundant at the beginning (Fig 3B), most populations from transfer 12 and all from transfer 20 were dominated by single *B*. *thuringiensis* strains (Fig 3C, S10 Table). These results are consistent with the idea that bacterial adaptation is commonly determined by strong clonal interference or clonal competition, subsequently leading to rapid fixation of single genotypes (reviewed in [32]). Alternative selective dynamics such as balancing selection, which would have led to the coexistence of several genotypes, do not appear to be involved. The results are thus also in contrast with those from our previous evolution experiment with the same interaction model which, in contrast to the current work, included pathogen immigration, and found an increase in pathogen genotype diversity under coevolution conditions [21,26]. These opposing findings are nevertheless consistent with previous modelling results that immigration can enhance diversity during coevolution [33,34].

At the strain level, we found that almost all coevolved and many one-sided adapted populations showed a high prevalence of BT-679 (Fig 3C), which is known to have stronger nematocidal effects than other pathogenic *B*. *thuringiensis* strains, such as BT-246 and BT-247, which are both present in the ancestral population [21,35]. Consistent with this observation, we found known nematocidal toxin genes to be almost exclusively restricted to the BT-679 genotype at transfers 12 and 20 and thus specifically enriched under coevolution and, to a lesser extent, one-sided adaptation conditions (Fig 4, S11–S13 Tables). This observation highlights the particular importance of toxin genes during the evolutionary interaction with a host, especially a coevolving host.

Interestingly, about half of the populations from the one-sided adaptation treatment are dominated at transfer 12 by the virulent, toxin-bearing BT-679, whereas the other half are dominated by the avirulent, toxin-lacking genotypes BT-22 and/or BT-50 (Figs 3C and 4). As indicated above, this result is most likely a consequence of the two phases that determine *B*. *thuringiensis* infection dynamics [22,23]. While the virulent genotypes are likely to have an advantage during the first phase (when the host is invaded and an infection is established), the avirulent genotypes that do not pay the cost of toxin production and/or plasmid replication are likely favoured during the second phase (when the host has already been weakened or killed) [22,23]. It seems that the nonevolving, ancestral host population used in the pathogen one-sided adaptation treatment produces a particular relative length of the two phases that favours both pathogen types to a similar extent over the entire infection cycle. Under these conditions, chance determines which of the two spreads to fixation, as observed across the one-sided adapted pathogen populations at transfer 12. Moreover, the spread of an avirulent genotype in some populations may then also explain the high extinction rate found for this treatment (Fig 1E). Only populations dominated by avirulent and toxin-lacking genotypes at transfer 12 were extinct at transfer 20, whereas all populations dominated by BT-679 at transfer 12 persisted until transfer 20 (Figs 3C and 4). Similarly, the additional population that went extinct at transfer 28 was dominated by an avirulent genotype at transfer 20 (replicate population 10 on the far right in row 4 of Figs 3C and 4). Consequently, the relative length of the two-phase infection process seems to determine extinction or persistence of pathogenic *B*. *thuringiensis*, whereby persistence is apparently enhanced in the presence of coadapting host populations, as available in the coevolution treatment.

### Specific Genomic Variants Are Selectively Favoured in the BT-679-Dominated Populations

**Fig 5:**
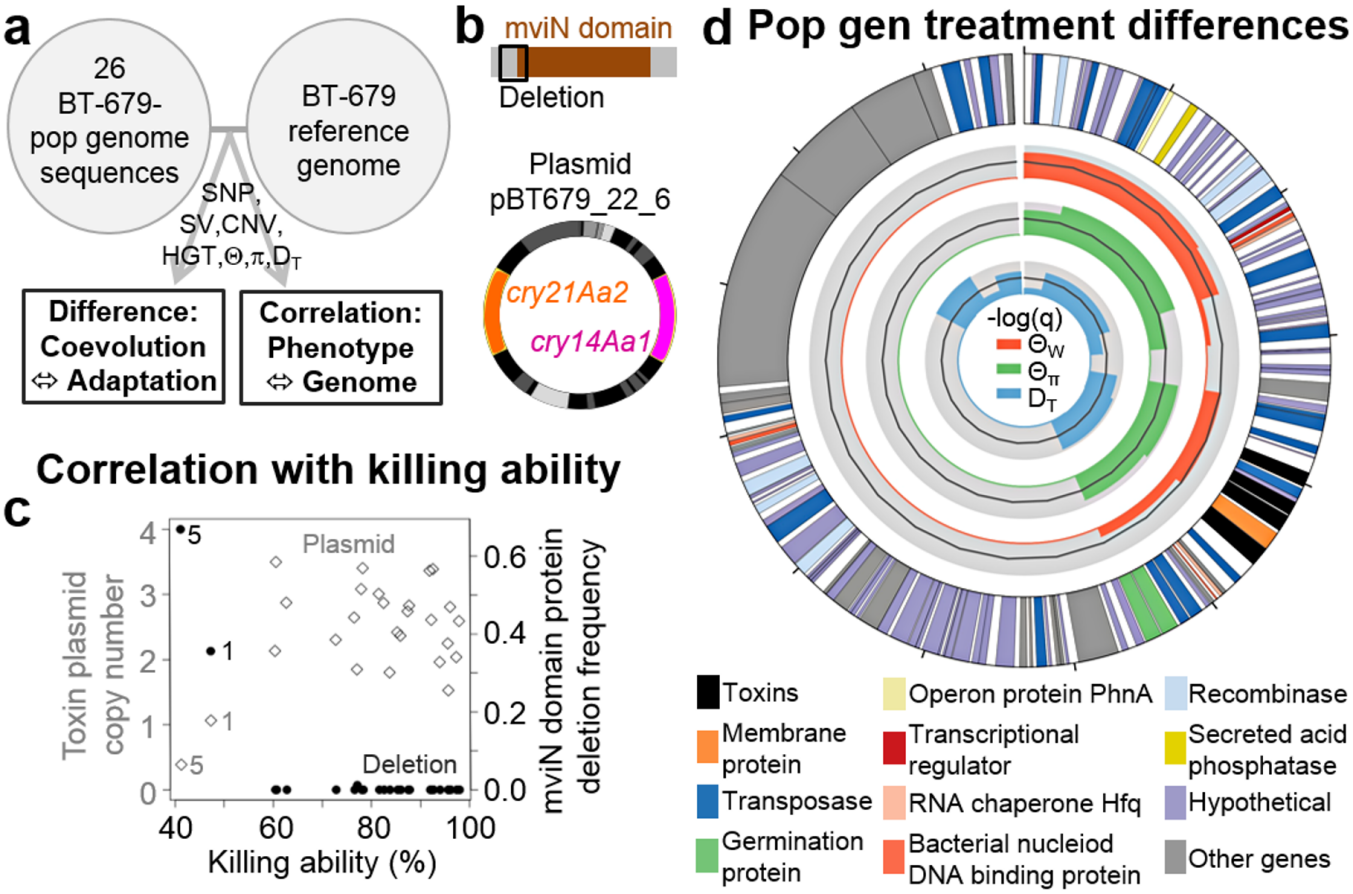
Fine-scale genomics and functional analysis demonstrate importance of nematocidal toxins and other genetic elements during adaptation. **A,** Workflow: Genomic variation of BT-679 populations was contrasted between treatments or correlated with phenotypic variation. **B,** mviN gene deletion and plasmid with cry toxins. **C,** Pathogen killing ability correlates negatively with mviN deletion frequency (left axis, filled circles) and positively with toxin plasmid copy number (right axis, open diamonds). The two most deviating values in all three considered traits were recorded for the same two populations (coevolved populations one and five, both from transfer 20, as indicated adjacent to the measured values), strongly indicating a link between reduced plasmid copy number, increased deletion frequency, and loss of virulence. **D,** Significant variation among the evolution treatments in population genomic statistics for the plasmid Bti_GWDALJX04I0LJH_51–405_fm319.5 (its structure is given in the outer circle). Using a sliding window-based analysis, approximately 65 kb of the plasmid yielded significant ANOVA FDR-corrected q-values, which are shown as-log_10_(q) on the light grey inner circles as coloured areas for the three inferred population genomic statistics (Θ_W_, Θ_π_, and D_T_; see Materials and Methods). The 5% significance threshold is indicated by the dark grey line within each light grey circle. Thus, coloured areas above this line indicate significant variation among the evolution treatments. This region contains genes encoding for transposases, toxins with unknown effect, a membrane protein, a secreted acid phosphatase, and other proteins (outer circle and legend at the bottom). The total size of the plasmid is about 126 kb. The original data is given in S5 Data, S19 Table, and S20 Table. The results for the statistical analysis is provided in S14–S21 Tables.

We next assessed whether specific genetic changes were selectively favoured within the 27 BT-679-dominated populations under coevolution and one-sided adaptation conditions. We established two novel complementary analysis pipelines to identify candidate regions under selection based on either: (i) comparisons between coevolution and one-sided adaptation treatments or (ii) correlations between genetic and associated phenotypic variations across all BT-679-dominated populations (see Materials and Methods, Fig 5A, S14–S20 Tables). Genetic changes were surveyed for single nucleotide polymorphisms (SNPs; measured through their individual frequency or their effect on population genetic statistics like θ_W_, π, and Tajima’s D), structural variations, sequence region copy number, and presence of horizontally transferred fragments (Materials and Methods) [36].

Based on our analysis pipeline, we identified more than 100 significant regions from the treatment comparison and four regions from the correlational analysis (S20 Table, Fig 5, and S5 Fig). The relevance of these candidate regions is difficult to assess because many only contain genes with unknown function. However, three of these regions harbour genes previously implicated in bacterial interactions with a host (see more detailed descriptions in Materials and Methods). One of these refers to an approximately 65 kb region of a large plasmid, for which population genetic measures in a sliding window-based analysis consistently indicate significantly higher variation under coevolution conditions. This plasmid contains putative host-interacting genes encoding toxins, a membrane protein, germination proteins, and an acid phosphatase (Fig 5D, S20 Table).

Variation in the two remaining regions correlated significantly with virulence. One of these regions encompasses a gene with unknown function that contains an mviN domain previously linked to virulence in different pathogens [37–39], and for which the frequency of a deletion correlates negatively with virulence (Fig 5B and 5C, S20 Table). The second region refers to a plasmid with two known nematocidal toxin genes, *cry14Aa1* and *cry21Aa2* [40], for which copy number positively correlates with virulence (Fig 5B and 5C, S20 Table). Copy number variation for this plasmid yielded one of the highest significance levels if compared with the other significant candidate regions (S20 Table), possibly emphasizing its particular relevance for the observed variation in killing ability. Intriguingly, the mviN gene deletion frequency also correlated negatively with the copy number of the toxin-containing plasmid. To reassess this, we performed two types of pairwise analyses of the two genomic variations and killing ability. The nonparametric Spearman's rank correlation test confirmed a significant relationship between the mviN gene deletion frequency and both plasmid copy number and killing ability but not between plasmid copy number and killing ability (S21 Table). However, all three comparisons produced significant associations when assessed with weighted regression analysis, for which plasmid copy number was weighted by the inverse of its variance to take into account the accuracy variations in the copy number estimates, which are proportional to the inferred coverage (S21 Table). Even though the associations for plasmid copy numbers are influenced by outliers, our analysis strongly suggests that the three traits covary. Thus, a decrease in virulence in some populations seems to coincide with an increase in mviN deletion frequency and a reduction in plasmid copy number (especially for the coevolved pathogen replicate populations one and five from transfer 20, highlighted in Fig 5C). This tripartite association may then imply that cry toxin abundance (as likely influenced by plasmid copy number) functionally interacts with an intact mviN domain-containing protein to determine virulence. Further functional analysis of this tripartite association, and especially the role of the yet uncharacterized mviN domain-containing gene, represents a particular challenge for the future.

### The Toxin Genes *cry14Aa1* and *cry21Aa2* Significantly Influence Pathogen Virulence

**Fig 6:**
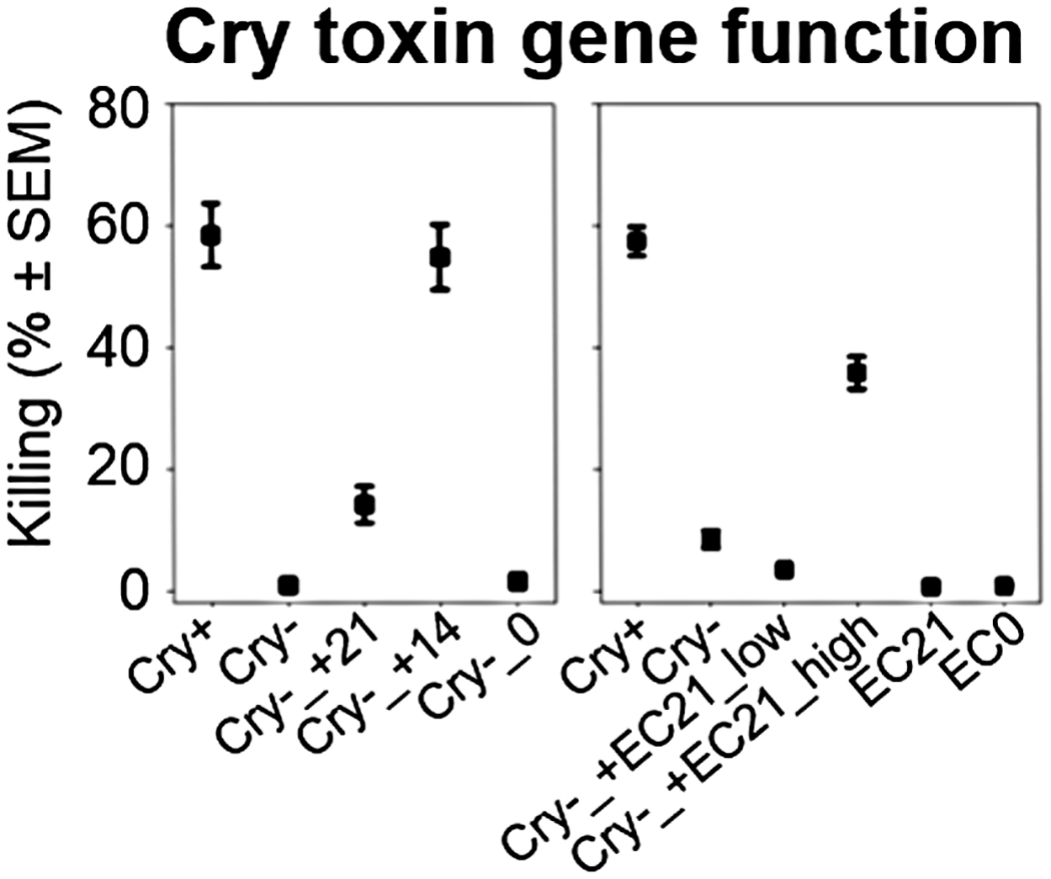
Virulence of BT-679 pathogens with or without nematocidal toxin genes. Mean virulence of plasmid-lacking BT-679 (Cry-) with reintroduced *Cry14Aa1* (+14) or *Cry21Aa2* (+21; left panel) or two concentrations of Cry21Aa2-expressing *E*. *coli* (+EC21_low, +EC21_high; right panel). Cry+, toxin gene plasmid-bearing BT-679; Cry-_0, empty vector control for BT-679; EC0, empty vector control for *E*. *coli*. The data is provided in S6 Data.

The nematocidal effects of the above implicated toxin genes *cry14Aa1* and *cry21Aa2* have previously been inferred from their heterologous expression in *E*. *coli* and subsequent exposure to *C*. *elegans* and other nematodes [40]. We thus asked whether their presence indeed explains the high killing ability of the strain BT-679, using a functional genetic approach based on a toxin-plasmid-lacking BT-679 variant (see Materials and Methods). Our analysis confirmed that loss of virulence in the toxin-plasmid-lacking variant (denoted Cry- in Fig 6) could be reconstituted to almost wildtype levels (denoted Cry+) by reintroduction of a plasmid with either of the two toxin genes (especially *cry14Aa1*, indicated by Cry-_+14 in Fig 6) or by addition of a high concentration of a Cry21Aa2-expressing *E*. *coli* (indicated by Cry-_+EC21_high in Fig 6; see also S22–S24 Tables). These results strongly suggest that the two toxin genes, and possibly their copy number, account for the nematocidal effects in BT-679 and may thus have been under positive selection under one-sided adaptation, and especially under during coevolution, where high virulence is particularly favoured by selection (Fig 1B, 1C, and 1F).

### Conclusion

To our knowledge, this is the first experimental evolution study that dissects the phenotypic and genomic consequences of coevolution rather than one-sided adaption for a bacterial pathogen, thus extending previous experiments, which were all exclusively based on bacteria–phage interaction models [16–20]. Our results highlight that coevolution particularly favours pathogen virulence, while distinct pathogen life history traits were selected for under one-sided adaptation (i.e., high infection load) or adaptation in the absence of a host (i.e., environmental persistence through biofilm formation). To our knowledge, this study is also the first to dissect the genetic basis of coevolutionary adaptation in a bacterial pathogen. Among the identified candidate genes, our analysis revealed a particular selective advantage of nematocidal toxin genes and their high copy number during the process of adaptation to a host organism, especially under coevolution conditions. Intriguingly, the selective advantage of high infection load under one-sided adaptation led to the loss of virulent and toxin-bearing *B*. *thuringiensis* genotypes in some populations, subsequently enhancing pathogen extinction. Our findings thus additionally suggest that the high levels of virulence often observed across *B*. *thuringiensis* natural isolates [41,42] may only be maintained if the target host is able to coadapt, indicating widespread coevolutionary interactions of *B*. *thuringiensis* and its various host taxa under natural conditions.

## Materials and Methods

### *C*. *elegans* and *B*. *thuringiensis* Material

The starting *C*. *elegans* host population was previously generated through consecutive crosses among 16 natural isolates (PB306, AB1, CB4858, CB4855, N2, JU400, MY16, JU319, PX174, MY1, PX179, JU345, CB4856, CB45507, RC301, and CB4852) [43]. These isolates cover the known worldwide genotype diversity for *C*. *elegans*. We adapted this genetically diverse population to our experimental conditions by maintaining it for ten generations at 19°C in 40 replicates in the presence of a nonpathogenic *B*. *thuringiensis* strain (DSM-350). This adaptation step served to minimize potential artifacts in the results of the main evolution experiment caused by predominance of environmental selection unrelated to the host–parasite interaction. These laboratory-adapted populations were mixed and cryopreserved in glycerol at −80°C in 200 aliquots (each containing approximately 5,000 worms) for later use in the main evolution experiment. Note that *C*. *elegans* larvae survive cryopreservation, thus allowing storage of worm populations for subsequent applications [44]. For all phenotypic experiments, hermaphroditic fourth instar larvae (L4) were used.

The starting pathogen population was similar to that used in our previous experiment [21] and consisted of a mixture of genotypes of the Gram-positive *B*. *thuringiensis*, including as dominant genotypes the strains MYBT18246 and MYBT18247 (both at an abundance of more than 10%; referred to hereafter and in the main text as BT-246 and BT-247, respectively) and also MYBT18679, MYBT22, and MYBT50 (less than 10% and more than 1%; referred to as BT-679, BT-22, and BT-50, respectively; see also Fig 3B of the main text). The host control treatment (see below) contained the non-nematocidal *B*. *thuringiensis* strain DSM-350. Prior to the evolution experiment, large quantities of *B*. *thuringiensis* cultures were prepared, aliquotted and conserved at −20°C for later use [21]. In all experiments, *B*. *thuringiensis* was used at a final concentration of 1.2 x 10^8^ particles/ml, always mixed with the standard *C*. *elegans* food source *E*. *coli* OP50 (final *E*. *coli* concentration of 2 x 10^9^ cells/ml).

### Experimental Evolution

The evolution experiment consisted of five treatments (Fig 1A): (i) host control, during which the host adapted to general laboratory conditions in the absence of pathogenic *B*. *thuringiensis*; (ii) host one-sided adaptation, where the host was allowed to adapt to a nonevolving pathogenic *B*. *thuringiensis* taken from a frozen stock culture at each transfer step; (iii) host–pathogen coevolution, in which both antagonists were continuously forced to coevolve with each other; (iv) pathogen one-sided adaptation, where the parasite was allowed to adapt to a nonevolving *C*. *elegans* population taken from a frozen culture at each transfer step; and (v) pathogen control, during which the pathogen adapted to general laboratory conditions in the absence of the nematode host. The treatment protocols for the five evolution conditions were otherwise completely identical.

In particular, the evolution experiment was run at a temperature of 19°C and included transfers to fresh media twice per week. Each treatment was run in ten replicates for a total of 28 transfers (equivalent to 14 weeks). Host population size was set to 500 individuals at each transfer step. 5% of the ancestral host population was added at every second transfer to simulate immigration in order to reduce the likelihood of drift effects. All treatments were maintained in wormballs, which we established as environments for *C*. *elegans*–*B*. *thuringiensis* coevolution experiments [21]. These consist of two halves of a transparent plastic ball, which are filled with a thin layer of the respective medium, followed by addition of bacteria and worms and subsequent closure of the halves [21]. The evolving host populations (treatments (i)–(iii) above) were purified and synchronized at every second transfer step with alkaline hypochlorite:NaOH, which is only survived by nematode eggs [44], thus eliminating any bacteria present [21]. The resulting eggs were raised to L4 larvae on NGM plates with *E*. *coli*, and then a total of 500 worms (475 evolved worms and 25 from the ancestral stock culture as immigrants) were transferred to the next round of the evolution experiment. Nematodes for the pathogen one-sided adaptation treatment were thawed at each transfer step from frozen aliquots and then raised as above before addition to the wormballs. For *B*. *thuringiensis*, the host-adapting populations (treatments (iii)–(iv)) were always isolated from dead worms, which were specifically collected at each transfer step and maintained for two additional days in phosphate buffered saline (PBS), followed by pasteurization at 80°C for 10 min to eliminate bacterial contaminants [21], subsequent culturing on NGM plates for 3–5 d, mixing with *E*. *coli* food, and transfer to the next selection round. *B*. *thuringiensis* for the host one-sided adaptation treatment were always taken from frozen stock cultures, and those from the pathogen control treatment were directly washed off the wormballs, followed in both treatments by pasteurization and all subsequent steps listed above. Samples from all replicate populations of all treatments were cryopreserved at transfers 12, 20, and 28. The general experimental protocol is similar to that used for our previous evolution experiments [21], and the exact methods for most of the host side of the experiment were recently published in Masri et al. [27].

### Phenotypic Analysis

Phenotypic changes across time and treatments were studied for the frozen host and parasite samples from transfer steps 0, 12, 20, and 28, using the same general environmental conditions as in the evolution experiment. These samples from the various transfer steps and treatments were characterized simultaneously and in random order to avoid artifacts because of observer bias and/or random environmental or temporal fluctuations. Both nematodes and bacteria were raised and purified prior to the experiments (alkaline hypochlorite:NaOH treatment for worms [44], pasteurization for bacteria). The hermaphroditic worms were used once they reached the L4 stage, and the final *B*. *thuringiensis* concentration was adjusted to 1.2 x 10^8^ particles/ml. We first assessed the presence of reciprocal coadaptations during experimental coevolution by comparing survival rate (see assay description below) of coevolved hosts exposed to coevolved pathogens from the same time point and replicate with those of coevolved hosts from the same replicate population exposed to ancestral pathogens and those of ancestral hosts exposed to coevolved pathogens from the same replicate. Thereafter, we analysed variation among evolution treatments for the evolved hosts and pathogens separately. To ensure comparability of evolved populations from the various treatments, we always exposed all evolved hosts or all evolved pathogens to their respective ancestral antagonist. For this analysis, several phenotypic traits were characterized, as described in more detail below.

Changes in host resistance sensu lato (i.e., the ability of the host to survive pathogen exposure) and pathogen virulence sensu lato (i.e., the ability of the pathogen to kill the host) were assessed by respectively measuring nematode survival and *B*. *thuringiensis* killing ability [21]. For this measure, 50 worms were exposed to *B*. *thuringiensis*, and the proportion of surviving hosts was counted after 48 h. Pathogen killing ability is simply represented by the inverse of this measure (i.e., the proportion of dead hosts). The results from these measurements are shown in Fig 1B, 1C, 1D, and 1F. As additional proxies of host resistance and pathogen virulence, we also examined the bacterium's effect on worm body size and population size [21]. For both traits, 35 L4 *C*. *elegans* were exposed to *B*. *thuringiensis*. After 48 h, body size was measured as whole worm area for four to six nematodes using differential interference contrast (DIC) microscopy (DM5000B microscope; Leica) and the program ImageJ 1.36b (http://rsb.info.nih.gov/ij/), followed by calculation of the average body size per replicate population for later statistical analysis. After five days of exposure, population size was determined by washing off all worms from the wormballs with 2 ml PBS, counting of animals in three 10 μl subsamples, and subsequent calculation of the total number of worms per replicate population. The results of these assays are shown in S1 Fig.

Infection load was quantified with a new protocol to characterize the ability of *B*. *thuringiensis* to ensure high abundance inside the host. For this assay, 35 worms were exposed to *B*. *thuringiensis*–*E*. *coli* mixtures (final concentrations respectively of 1.2 x 10^8^ particles/ml and 2 x 10^9^ cells/ml) on PFM plates. After 48 h, three to six live worms per replicate were transferred onto a 12 well microscopic slide, followed by body size measurements using ImageJ 1.36b. To remove bacteria adhering to the cuticle, the worms were carefully washed with approximately 20 μl sterile H_2_O under a dissecting microscope, followed by their transfer into 1.5 ml tubes containing 100 μl H_2_O. The number of externally associated bacteria, which could not be removed, was estimated by counting cells in the surrounding solution using standard Thoma counting chambers (0.1 mm depth). For each replicate, bacteria were subsequently extracted by sonicating the worms for 10 sec, 6 cycles at 60 Hz, followed by addition of four 1 mm Zirconia beads and vortexing for 3 sec. The number of extracted bacteria was quantified using Thoma chambers. The infection load was then calculated as the number of extracted bacteria minus the number of bacteria in the surrounding solution, adjusted for worm size and averaged per replicate population. The results are shown in Fig 1G.

The characteristics of biofilm formation were studied for all of the evolved replicate populations, and, for a separate set of assays, a selection of isolated clones from these populations. All evolved replicate populations from transfers 0, 12, 20, and 28 were characterized with two assays: (i) as a rough qualitative proxy of biofilm formation, we scored the proportion of replicate populations per treatment that produced clearly visible flakes (Fig 2A and 2B); (ii) biofilm formation was quantified by measuring average particle size produced by each replicate population. 20 μl per population were grown for 96 h at 19°C on NGM, washed off with 3 ml PBS, vortexed for 5 sec in 15 ml tubes, followed by measuring particle area (in mm^2^) for the five largest particles within a random 20 μl sample of the culture using DIC microscopy and ImageJ. Four random 20 μl samples were assessed per replicate and averaged for subsequent statistical analysis. The results of this assay are shown in Fig 2D.

An additional analysis of the dynamics of biofilm formation was performed for four individual clones, isolated from transfer 20 from above selected populations. We confirmed that the isolated clones showed the same general characteristics as their source populations (i.e., one highly virulent, non-biofilm-forming clone from the coevolution treatment; one highly virulent, non-biofilm-forming clone from the one-sided adaptation treatment; one non-nematocidal, biofilm-forming clone from the one-sided adaptation treatment; and one non-nematocidal, biofilm-forming clone from the control). The dynamics of biofilm production were characterized by growing the selected four clones, as well as three of the ancestral strains (BT-246, BT-247, and BT-679), on 9 cm NGM plates in several replicates. Every 24 h, an entire plate was washed off for particle size measurements as described above. The entire analysis was performed for a total of 144 h (Fig 2C).

Moreover, these four clones were further characterized by assessing their bacterial competitive ability under either low nutrient conditions on PFM or high nutrient conditions on NGM. Biofilm-forming and non-biofilm-forming bacteria (concentration of 1.2 x 10^9^ particle/ml) from the selected evolved clones were streaked out along thin lines in parallel to each other at a distance of 5 mm and grown at 19°C for 96 hours on NGM and 21 days on PFM (due to the absence of nutrition, growth was substantially reduced under these conditions). Thereafter, the growth expansion of one clone in the direction of the other clone was measured as the distance from the original streak to the farthest area of the grown culture. An analogous measurement was taken for the competing bacterium. A competitiveness index was subsequently calculated for a particular clone by taking its growth expansion measurement and subtracting from it the respective measurement of the competitor. Thus, a competitiveness index of 0 indicates equality, whereas a positive index suggests higher competitiveness for the focal population (Fig 2E).

Statistical analysis of phenotypic data was based on JMP 9 (SAS). Variations between the treatments in almost all traits (exceptions are given below) were evaluated with a general linear model including transfer, treatment, and the interaction between the two as fixed factors and replicate population as a random factor nested within the treatment factor. For the analysis of reciprocal coadaptations, we used a general linear model based on ordinal logistic regression, using exposure type and replicate as factors. A factor effect test was used to assess the relative influence of the defined factors in the model, as implemented in JMP 9 (SAS). Variation in competitiveness was compared with the Mann-Whitney U test (MWU). Variation in the number of replicate populations per treatment and time point, which are able to produce biofilm flakes (qualitative assessment of biofilm formation), was assessed with a Fisher exact test. Graphs were generated with SigmaPlot version 11.0 (Systat Software Inc.). The results of the statistical analysis of the phenotypic data are shown in S1–S7 Tables.

### Genome Sequencing

Draft genome sequences for five *B*. *thuringiensis* strains present in the starting population (BT-246, BT-247, BT-679, BT-22, and BT-50) were used as references for mapping of the population genomic data. For each of these strains, genomic DNA was isolated using a DNeasy Blood and Tissue Kit (Qiagen). Whole genome sequencing was performed using the Roche 454 Genome Sequencer FLX platform. The resulting reads were assembled using GS *De Novo* Assembler (Roche). For MYBT18679, a partially closed reference was generated through targeted PCR and Sanger sequencing, consisting of 31 scaffolds, including more than ten plasmids. A summary of the data and assemblies for each strain is shown in S8 Table.

For samples of the ancestral population and each of the evolved replicate populations from transfer 12 and 20, genomic DNA was isolated following the Qiagen DNeasy Blood and Tissue kit procedures for gram-positive bacteria. Prior to DNA extraction, 10 μl of the frozen bacterial populations were spread onto NGM plates and grown for 14–16 h at 25°C. Bacteria were washed off plates with 1 ml of autoclaved H_2_O, followed by DNA extraction. For samples showing the biofilm phenotype, four replicates were extracted and pooled, while three replicates were extracted for the other samples. DNA quantity, measured with Qubit Fluorometric Quantitation, ranged between 9.13 ng/μL and 55.1 ng/μL. For Illumina sequencing, genomic paired-end libraries were prepared following standard methods [45]. Insert sizes (excluding adapters) ranged from 200–450 nucleotides. Libraries were sequenced using GAII or GAIIx Illumina sequencing instruments to yield paired 100mers. The Illumina image analysis pipeline with default parameters was used for image analysis, base-calling, and read filtering. Further filtering served to remove adapter and PhiX contamination based on blast alignment (pairs with ≥ 14nt aligned at ≥ 98% were removed). The reads were subsequently processed with SeqPrep (https://github.com/jstjohn/SeqPrep) software to remove adapter sequences and merge overlapping read pairs. The raw read data are available from the ENA database (ENA; www.ebi.ac.uk/ena/) under study number PRJEB5931.

### Comparison of Mapping Software

We assessed suitability of mapping software programs (Bowtie [46]; BWA [47]; MOSAIK [48]; SOAP [49]; and GSNAP [50]) to correctly align Illumina reads from population samples (thus including nucleotide and structural variation) to our concatenated metareference. We first simulated reads from three of the publicly available *B*. *thuringiensis* genome sequences (Genbank accession number NC_014171.1, NC_005957.1 and NC_008600.1) using the dwgsim tool from the dnaa 0.1.2 software suite (http://sourceforge.net/projects/dnaa/). A depth of coverage of 1x for each genome was generated corresponding to 55,000 reads of 100 bp with a fragment size of 350 bp per genome and maximum quality. A metareference was generated by aligning the five genomes using progressiveMAUVE [51] and polymorphic sites were recorded following ambiguity IUPAC codes to avoid counting them as mismatches in the alignment. For GSNAP, a SNP file was created to account for variation. Indel positions were kept without gaps. Usage of SOAP and Bowtie led to low mapping efficiency (S3 Fig), apparently because of imprecise alignment of polymorphic positions. MOSAIK and GSNAP performed equally well, while BWA aligned substantially fewer reads to the metareference (S3 Fig). Based on these results, SOAP and Bowtie were excluded from subsequent analyses.

A second set of simulated data was generated to test the influence of allele frequency biases, which are likely to be present in the evolved populations. Ten genomes of each of the three references were generated with SNP variation, resulting in a total of 30 different genome sequences that were simulated as 100 bp paired-end reads with 1,000x read depth. These produced the site frequency spectrum shown in S2 Fig. The reads were mapped with the three programs, followed by detection of SNPs using SNVer [52] and allele frequency calculations based on the number of SNP reads divided by the total read depth. Based on this data set, which is likely to be representative of the sequence data from our evolved populations, we found GSNAP to produce a site frequency spectrum most similar to the original distribution (S4 Fig). GSNAP was therefore used as mapping software.

### Broad-Scale Genome Analysis: Strain Composition of Evolved Populations

Our strategy for estimating the frequency of the five ancestral strains in pooled population samples consisted of four steps (Fig 4A). We first generated a concatenated metareference based on the five ancestral strains. Secondly, the obtained reads of the considered population samples were mapped onto the metareference using GSNAP, resulting in 90%–97% mapping efficiency (S8 Table). Thirdly, we identified the polymorphic sites where only one of the five reference strains shows a substitution. For the population samples, we then determined the frequency of substitutions at each of these diagnostic polymorphic positions and took these as independent estimates of strain frequencies (S9 Table). Fourthly, as such frequency distributions are usually asymmetric (e.g., left-skewed), we calculated the mode of the distribution as the final frequency estimate, using the function mlv from package modeest on the R platform [53]. The results of the statistical comparison among the evolved populations are given in S10 Table, and the inferred values per strain are presented in Fig 3.

### Fine-Scale Genome Analysis of BT-679-Dominated Populations: Variant Detection

For the fine-scale genome analysis, we focused on the evolved populations dominated by the BT-679 strain. These populations still showed substantial variation in both killing ability and infection load. The analysis was based on a four-step strategy (Fig 5A). Firstly, each read was mapped to each of the five reference genomes present in the starting population. Secondly, the edit distance between the reference and the mapped read (NM field in SAM format) was recorded and compared among the five alignments (referring to the five reference genomes). Only reads that produced the lowest edit distance to the BT-679 genome were considered for further analysis (i.e., they had the highest similarity to BT-679). Thus, reads with the same or lower edit distance to the non-BT-679 strains were excluded (see mapping statistics in S9 Table, where reads mapping uniquely to each reference and total reads mapped are reported). Thirdly, SNVer software version 0.4.1 [52] was used to identify SNPs and short indels using default parameters, except that the strand bias and the Fisher’s exact test threshold was set to 20 instead of 30 (-u 20) to avoid frequency bias due to overfiltering [54]. Minimum mapping quality and base quality were set to 20, and the results are shown in S14 Table. Further filtering consisted of: (i) excluding positions for which an identified SNP was below the 2% or above the 98% quantile of the observed coverage distribution; (ii) excluding SNPs and short indels if a significant Fisher’s exact test on strand bias was inferred (0.05 threshold); (iii) excluding SNPs if an indel is detected at the same position; and (iv) keeping SNPs with a minimum allele frequency (MAF) across the sample above 5%. Finally, we identified structural variations using Pindel version 0.2.4 [55] with default parameters except the following: −w 1 (1 million base bins) and −u 0.03 (maximum allowed mismatch rate). The results are summarized in S15 Table.

### Fine-Scale Genome Analysis of BT-679-Dominated Populations: Copy Number Variation and Horizontal Gene Transfer

Several tools have been developed to detect copy number variations (CNVs) using depth of coverage (e.g., CNVnator [56] or Event Wise Testing [57]). However, these approaches have not been designed to account for pooled population samples where only some individuals may harbor a CNV, possibly leading to only a proportional coverage change below but not above the value of one. Therefore, we developed our own approach. Firstly, we used the average rank of each position instead of the raw or scale data in order to account for general coverage variations among samples. Secondly, we calculated the variance at each position for the rank of the depth of coverage across the samples. Thirdly, outliers were extracted using the getOutliers() function of the extremevalues package on the R platform with the method I and a normal fit. Adjacent outlier positions (i.e., with a distance of less than 100 bp) were considered to belong to the same coverage singularity. Fourthly, scale coverage relative to the median coverage of chromosomal contigs was calculated at candidate position to estimate the average copy number in each sample (S16 Table).

Following a similar approach, we also assessed copy number variation for each contig within the BT-679 reference by calculating the ratio of each contig over the average of all chromosomal contigs. The variance was estimated by random sampling of 10,000 positions. The results are presented in S17 Table.

Horizontal gene transfer (HGT) was evaluated by identifying non-BT-679 genome regions within the populations dominated by BT-679. For this, we extracted reads mapping uniquely and best to one of the non-BT-679 reference genomes. We only considered the thus identified putative HGT fragments, for which an indication of HGT from the same reference genome is continuously found across at least 1 kb. The frequency of each putative HGT was then estimated through the ratio of the median coverage of the fragment over the median coverage of the chromosomal contigs of BT-679 (S18 Table).

### Fine-Scale Genome Analysis of BT-679-Dominated Populations: Population Genetic Analysis

We calculated three different population genetics statistics in a sliding window approach with 5 kb steps and 10 kb window size, namely Watterson’s θ, Tajima’s π, and Tajima’s D. To correct for coverage variation within a window and along the genome, coverage was taken as a proxy for the number of samples, and statistics were calculated using the adjusted Watterson’s θ and Tajima’s π estimates that specifically allow for sample size variation across the genome [36]. Only polymorphisms showing a frequency above 0.05 were considered. The results are summarized in S19 Table.

### Fine-Scale Genome Analysis of BT-679-Dominated Populations: Statistical Analyses

The same statistical tests were performed on each dataset, which either contained the identified SNPs, short indels, pindel structural variants, CNVs detected through coverage variation, putative HGTs, or the population genetic characteristics. We excluded coevolution replicate 3 at transfer 12 from the analysis because it contained two genotypes at higher frequencies (BT-679 and BT-22), and it was thus not directly comparable to the other replicate populations dominated by BT-679. Two types of statistical analyses were performed. Firstly, a linear regression analysis was performed using genomic variation versus either killing ability or infection load. The linear regression (using R [53]) was weighted by the log_10_(coverage) on each dataset except of the population genetics statistics, because the read depth coverage is directly affecting the variance on frequency estimates. Secondly, an ANOVA was performed to compare the difference between treatments. The treatment effect was nested within transfer as follows:

Variable~Transfer+Treatment[Replicate]+Transfer*Treatment[Replicate]

Significance levels were adjusted using the FDR [28].

The statistical analysis identified a large number of genome regions (S20 Table). At least some of them, but possibly not all, may have influenced pathogen adaptation to either coevolving or nonchanging host. In order to identify the most relevant regions for such adaptive processes, we used the following statistical and functional criteria: (i) the relevant genome regions should have been identified through variation in at least four replicate populations (and thus not be the consequence of exceptional events in very few populations; note that under the latter conditions, homoscedasticity of the data—as required for ANOVA—may also be compromised); (ii) for the ANOVA approach, they should only show a treatment effect and not a transfer or an interaction effect, the latter of which may both indicate convergent evolution across treatments during the course of the experiment; (iii) for the analysis of horizontal gene transfer, treatment variance of the transferred region should exceed 0.04; and (iv) the identified variations should be of functional consequence; for example they should influence gene expression levels (i.e., changes in copy number) or directly influence gene function (nonsynonymous or frame-shift mutations, etc). The resulting list of candidate regions is presented below and highlighted in yellow and bold font type in S20 Table.

Two of the identified regions were found to covary with killing ability: the deletion frequency of the mviN virulence gene and the copy number of the cry toxin-containing plasmid. This relationship was reassessed through pairwise analysis of the three characteristics using two approaches: (i) Spearman's rank correlation analysis and (ii) weighted regression analysis, whereby plasmid copy number was weighted by its variance to reduce the influence of extreme values.

### Fine-Scale Genome Analysis of BT-679-Dominated Populations: Overview of Identified Genome Regions

The linear regression analysis revealed four cases of a significant association between genome and killing ability but none with infection load. Three of the four significant cases may be of relevance for bacterial adaptation to either coevolving or nonchanging host as they could have functional consequences (i.e., they affect expression levels of genes or gene function itself). In particular, killing ability was found to correlate positively with the copy number of (i) the plasmid pBT679_22_6, which contains the nematocidal toxin genes *cry21Aa2* and *cry14Aa1* (q-value = 1.73E-07; Fig 5B and 5C); and (ii) the plasmid (or plasmid fragment) represented by the contig Bti_GWDALJX04IG4JR_1–226, containing a plasmid recombination enzyme, two hypothetical proteins and Parvulin-like peptidyl-prolyl isomerase (q-value = 0.016). Killing ability also correlated negatively with the frequency of a chromosomal deletion of 12 amino acids in a putative virulence factor containing an mviN domain (gene Bt_01995; q-value = 0.038; Fig 5B and 5C). Taken together, the region with the strongest effect (according to the q-value) refers to the plasmid that contains genes with known nematocidal effect and that is thus known to have a function in pathogen–host interactions. Interestingly, copy number of this plasmid and the mviN domain-containing protein deletion frequency not only correlate with killing ability but also with each other (Fig 5C, S21 Table). Here, the significant negative correlation indicates that a high protein deletion frequency coincides with a low plasmid copy number in the same replicate populations, suggesting a functional relationship between these.

The ANOVA approach yielded a comparatively large list of genome regions with a significant treatment effect, strongly suggesting that the imposed differences in selection conditions lead to changes in the favoured genomic variants and/or promoted horizontal gene transfer. In particular, a total of 53 genome regions were inferred from the SNP analysis, 3 from the Pindel-based structural analysis, 81 from the coverage-based copy number variation analysis, 66 from the assessment of horizontal gene transfer, and 35 from the population genetic analysis. Note that some of these regions overlap as a consequence of the different approaches used during the respective analyses. Based on the above outlined conservative criteria, only a few of the identified regions are likely of relevance for adaptation to either coevolving or nonchanging hosts (S20 Table), including (i) a recombinase or invertase (gene Bti_05865), for which the gain of a stop codon varies among treatments (inferred through SNP analysis); (ii) a predicted acetyltransferase or hydrolase (gene Bti_05100), which shows copy number variation between treatments; (iii) two horizontally transferred gene regions from the *B*. *thuringiensis* strain BT-246 containing a Cysteine protease, a recombinase or invertase, and several hypothetical proteins; (iv) one horizontally transferred gene region from BT-247, containing among others a transcriptional antiterminator; (v) one horizontally transferred 16S rRNA gene region from BT-50 (S5 Fig); and (vi) an approximately 65 kb region from the plasmid contig Bti_GWDALJX04I0LJH_51–405_fm319.5, consistently identified by the population genetic measures to vary among treatments and containing a variety of different genes such as those encoding toxins (with unknown effects), a membrane protein, a transposase, germination proteins, a secreted acid phosphatase, and others (Fig 5D). None of the above regions contains genes previously implicated in the bacterium's interaction with a host. The only exception may refer to some of the genes found in the 65 kb plasmid region, of which the toxin, the membrane protein, the acid phosphatase, or the germination protein genes could be speculated to contribute to host interactions. The dissection of the above genes' exact role in shaping adaptation to either coevolving or nonchanging hosts represents a particular challenge for future research.

Interestingly, 14 of the inferred cases of copy number variations refer to collagen triple helix repeats (S20 Table), possibly suggesting a role of these genes in general adaptation to a host environment, irrespective of whether the host is coadapting or not. It is similarly interesting to note that horizontal transfers mainly originated from two ancestral non-nematocidal *B*. *thuringiensis* strains that are mainly, yet not exclusively, favoured in the absence of the host. Of these, most transfers came from strain BT-22, encompassing 35 horizontally transferred fragments with a total length of 51 kb; whereas 19 fragments with a total length of 45 kb originated from BT-50. One of the transferred fragments refers to a phage that originated from BT-50 and spread through the BT-679-dominated populations across time, irrespective of the evolution treatment regime and possibly as a selfish element that does not contribute to bacterial adaptation to a host (S5 Fig).

### Toxin Gene Screen

To identify genes for crystal toxin proteins that were present in the starting population of *B*. *thuringiensis*, we performed sequence similarity searches on the draft genome assemblies of the nematocidal strains BT-246, BT-247, and BT-679 using known cry toxin protein sequences as queries. The query sequences were derived from the cry toxin list available on the Bt toxin nomenclature webpage (http://www.lifesci.sussex.ac.uk/home/Neil_Crickmore/Bt/). Based on this analysis, we identified seven genes with high similarity to known cry toxin sequences: *cry13Aa1* in BT-246, *cry6Ba1* in BT-247, and *cry14Aa1*, *cry21Aa2*, *cry34Aa4*, *cry35Aa4*, and *cry38Aa1* in BT-679. *cry14Aa1* and *cry21Aa2* are located 8 kb apart on a 23 kb plasmid, while *cry34Aa4*, *cry35Aa4*, and *cry38Aa1* are all located within a 4 kb region on a separate plasmid. We also identified several additional putative cry toxin genes (<60% similarity to query sequences), but for practical reasons they were not considered in subsequent analyses.

To analyse the composition of crystal toxin genes in the evolved *B*. *thuringiensis* populations, we focused on *cry13Aa1*, *cry6Ba1*, *cry14Aa1*, *cry21Aa2*, and *cry35Aa4*. Twenty individual clones were isolated from each available evolved population from transfer 12 and 20 by plating the population on nematode growth medium (NGM) plates and picking single colonies, resulting in a total of 1,100 clones tested. The clones were grown overnight at 28°C in LB medium and then frozen at -20°C. This frozen material was used directly in PCRs with toxin-specific primers (S11 Table) and 15.6 μl reaction volumes containing 0.39 units GoTaq DNA Polymerase (Promega), 1x Green GoTaq reaction buffer, 0.2mM each dNTP, and 0.4 μM of each primer. Thermal cycling was performed with an initial denaturation step at 95°C for 2 min followed by 35 cycles of 30 sec 95°C, 30 sec 57°C, 90 sec 72°C, and then a final extension at 72°C for 10 min. *CodY* primers were included in each reaction to ensure integrity of the template. We additionally determined the chromosomal background of each clone by Sanger sequencing of part of the *codY* gene, amplified by PCR as above.

The chromosomal backgrounds were largely consistent with the whole genome data (Fig 3). The coevolution treatment was dominated by BT-679 and the control treatment by BT-22, while the adaptation treatment showed variation between replicates with virulent populations dominated by BT-679 and nonvirulent populations dominated by BT-22 or BT-50 (S12 Table). The toxin genes *cry14Aa1*, *cry21Aa2*, and *cry35Aa4* were only found in evolved BT-679 clones, thus remaining within the same chromosomal background. Their presence varied among these clones, whereby c*ry14Aa1* and *cry21Aa2* showed the same pattern (i.e., both present or both absent) for all but seven clones and were both less abundant than *cry35Aa4* (Fig 4). *cry13Aa1* was found only once in a BT-246 background, while *cry6Ba1* was absent, consistent with the very low abundance of BT-246 and BT-247 in the evolved material. The distribution of the BT-679 toxin genes differed significantly between coevolution and control conditions and between some of the coevolved and one-sided adapted populations (Fig 4, S13 Table).

### Functional Analysis of Toxin Genes

We used two complementary approaches to assess the nematocidal effect of cry toxin genes from BT-679. On the one hand, we expressed one of the toxin genes, *cry21Aa2*, in *E*. *coli*, followed by *C*. *elegans* survival analysis. On the other hand, we introduced either *cry14Aa1* or *cry21Aa2* into a BT-679 variant that lost the 22.5 kb plasmid carrying these two toxin genes (denoted BT-679_Cry-), again followed by analysis of nematode survival.

For the former approach, the entire coding region of *cry21Aa2* was amplified by PCR (see below) and cloned into the expression vector pQE30 using standard procedures. Both the pQE30 with *cry21Aa2* and the empty vector were transferred into *E*. *coli* JM109 by electroporation and selection on ampicillin-containing medium (100 μg/ml). Prior to nematode survival experiments, *E*. *coli* was cultured at 37°C overnight in LB medium, containing ampicillin (100 μg/ml) and IPTG (200 μg/ml). The bacteria were washed twice and cell density was adjusted to OD_600_ = 5. Virulence of the resulting *E*. *coli* strains was assessed using exactly the same methods as described above for phenotypic analysis of the evolved material (chapter 1.3). The main exception was that we used an isogenic *C*. *elegans* strain (the standard laboratory strain N2) and standard Petri dishes instead of wormballs. Nematode survival was assessed after 48 h under six treatment conditions: (i) the ancestral BT-679 with cry toxins (BT-679_Cry+); (ii) the BT-679_Cry− strain lacking the two tested toxin genes; (iii) BT-679_Cry− combined with a low concentration of the *cry21Aa2*-expressing *E*. *coli* (a 1:10 dilution of the OD5-concentrated stock); (iv) BT-679_Cry− combined with a high concentration of *cry21Aa2*-expressing *E*. *coli* (the OD5-concentrated stock without any dilution); (v) only the *cry21Aa2*-expressing *E*. *coli* (at the OD5 stock concentration); and (vi) only the empty vector *E*. *coli* strain. In all cases, the empty-vector *E*. *coli* strain was added as food.

For the second approach, we first substituted *gfp* with a multiple cloning site (MCS) in the pHT315 p*AphA*-*gfp* plasmid that is used as an *E*. *coli*–*B*. *thuringiensis* shuttle vector (kindly provided by Christina Nielsen-LeRoux, Guyancourt, France). For this, the MCS of the pUC19 plasmid (Carl Roth, Germany) was amplified by PCR using Phusion High-Fidelity DNA polymerase (Thermo Scientific, Germany) and primers MCS_f and MCS_r (S21 Table). The PCR product was gel-purified (QIAquick Gel Extraction Kit and PCR purification kit, both Qiagen, Germany), digested with *Hin*dIII and *Xba*I, ligated with T4 DNA ligase (Thermo Scientific, Germany) into the respective sites of pHT315_p*AphA-gfp* to create pHT315_p*AphA-*MCS. This vector was introduced into *E*. *coli* Top10 (Invitrogen, US), grown in LB with ampicillin (100 μg/ml), and followed by plasmid isolation (QIAprep Spin Miniprep Kit, Qiagen, Germany). Thereafter, the entire coding regions of *cry21Aa2* and *cry14Aa1* were PCR-amplified using Phusion High-Fidelity DNA polymerase (Thermo Scientific, Germany) and the respective primers (S21 Table), digested with *Sal*I and *Pae*I, ligated into pHT315_p*AphA-*MCS, followed by transformation into *E*. *coli* Top10 and plasmid isolation as above. *B*. *thuringiensis* BT-679_Cry- was transformed with three different vectors (containing either *cry14Aa1*, *cry21Aa2*, or the red fluorescent protein (*rfp*) as a control), using electroporation with a Bio-Rad Gene Pulser (Bio-Rad, Germany), as described previously [58]. Transformants were grown in LB containing erythromycin (10μg/ml), and presence of the correct inserts was confirmed by Sanger sequencing. Prior to survival experiments, the *B*. *thuringiensis* strains were grown for four days at 19°C on NGM, washed in S buffer, and had the concentration adjusted to 1.2 x 10^8^ particles/ml, generally following the procedures outlined above for the evolution experiment. The survival experiment was performed using the same methods as above, including the N2 *C*. *elegans* strain and Petri dishes for exposure. The empty-vector *E*. *coli* strain was always added as food. Survival was tested for a total of five treatments: (i) the ancestral BT-679 containing all cry toxins (BT-679_Cry+); (ii) the BT-679_Cry- lacking the two toxin genes; (iii) the BT-679_Cry-, which contains the *cry14Aa1*-expressing plasmid (BT-679_Cry-_+14); (iv) the BT-679_Cry-, which contains the *cry21Aa2*-expressing plasmid (BT-679_Cry-_+21); and (v) and the BT-679_Cry- with the *rfp*-expressing plasmid as a control (BT-679_Cry-_0).

The results of the two assays are shown in Fig 6, and the statistical results are given in S23 and S24 Tables

## Supporting Information

S1 Data Results on phenotypic changes in both host and pathogen during experimental evolution, including data for reciprocal adaptations of coevolved hosts and pathogens, changes in evolved hosts exposed to ancestral pathogens (survival, population growth, body size, and infection load), and changes in evolved pathogens exposed to ancestral hosts (killing ability, infection load, pathogen effect on host population growth and host body size). The data are summarized in Fig 1, and S1 Fig, and the statistical results are shown in S1–S5 Tables.
(XLSX)

S2 Data Results on the analysis of biofilm formation in evolved *B*. *thuringiensis*, including frequency of evolved populations able to form biofilms, quantitative analysis of particle size across time for specific bacterial clones, mean particle size for evolving *B*. *thuringiensis* populations, and competitive ability of either biofilm-forming or non-biofilm-forming clones on either nutrient-rich or nutrient-poor medium. The results are summarized in Fig 2, and the statistical results are given in S6 and S7 Tables.
(XLSX)

S3 Data Results on the relative frequencies of *B*. *thuringiensis* strains in the evolving populations. A summary of the results is shown in Fig 3, and the statistical results are given in S10 Table.
(XLSX)

S4 Data Results on the frequency of *B*. *thuringiensis* BT-679 cry toxin gene combinations in the evolving populations. The results are shown in Fig 4 and the statistical results given in S13 Table.
(XLSX)

S5 Data Data on the relationship between killing ability, frequency of the mviN deletion, and copy number of the cry toxin plasmid, and also data on the annotation of the plasmid showing significant population genetic differences among evolution treatments. The results are summarized in Fig 5, and the statistical findings are shown in S14–S21 Tables.
(XLSX)

S6 Data Original data for virulence of BT-679 pathogens with or without nematocidal cry toxin genes. The results are shown in Fig 6. The statistical analysis is given in S23 and S24 Tables.
(XLSX)

S7 Data Results on the efficiency of read mapping by different software programs. The results are shown in S3 Fig.
(XLSX)

S8 Data Results on site frequency spectrum of simulated genomes, as illustrated in S4 Fig. (XLSX)

S9 Data Results on two cases of horizontal gene transfer, as highlighted in S5 Fig. (XLSX)

S1 Fig Variation among evolved pathogen populations from different treatments (colors as in Fig 1A) upon exposure to the ancestral host. **A**, Variation in the pathogen's effect on host population size; and **B**, in the pathogen's effect on host body size. Bars show standard errors. S4 Table and S5 Table show the corresponding statistical results. The original data is provided in S1 Data.
(TIF)

S2 Fig Electron micrograph of a solid and highly robust biofilm particle produced by control-evolved *B*. *thuringiensis*. (TIF)

S3 Fig Analysis of five mapping programs as to their ability to correctly align simulated reads from *B*. *thuringiensis* genomes. The five mapping programs are given along the *x*-axis. The *y*-axis presents the total number of reads mapped, classified in four categories following the samtools flagstat function: (i) reads unmapped (yellow bar area); (ii) not properly paired: both reads of a pair are mapped onto the reference genome but expected insert size and/or orientation is incorrect (red bar area); (iii) singletons: only one read of the pair is mapped (light blue area); and (iv) properly paired: both reads are mapped onto the reference genome with correct orientation and expected insert size (dark blue area). The data is shown in S7 Data.
(TIF)

S4 Fig Site frequency spectrum of 30 simulated *B*. *thuringiensis* genome sequences derived from three reference genomes. Original spectrum relative to the reference genome NC_014171.1 (expected results on far left), and the results obtained with the mapping software BWA, MOSAIK, and GSNAP. The data is shown in S8 Data.
(TIF)

S5 Fig Exemplary cases of horizontal transfers with significant variation between treatments or transfers. **A,** Significant variation among treatments for a 16S rRNA gene, horizontally transferred from the BT-50 ancestral strain to the BT-679 genotype. **B,** Horizontal transfer and spread of a phage from the non-nematocidal ancestral strain BT-50 in the BT-679 coevolved and one-sided adapted populations across time. The different replicate populations are given along the *x*-axis and DNA fragment frequency on the *y*-axis. Red indicates coevolution and green one-sided adaptation. The data is shown in S9 Data.
(TIF)

S1 Table Analysis of reciprocal coevolutionary adaptation in comparison to adaptation to the ancestral antagonist. Pairwise comparison of the different exposure types. Here, the coevolved–coevolved exposures from a particular replicate population were compared with the corresponding exposures, in which the same coevolved host or pathogen replicate was confronted with the ancestral antagonist. Significant values after FDR adjustment are given in bold. The data is provided in S1 Data.
(DOCX)

S2 Table Comparison between evolved and ancestral host phenotypes. Comparison between evolved (host coevolution, host one-sided adaptation, and host control) and ancestral hosts both exposed to ancestral pathogens using an analysis of variance. Degrees of freedom (df) are given for the comparison and the error (before and after comma, respectively). Significant values after FDR adjustment are given in bold. The data is provided in S1 Data.
(DOCX)

S3 Table Analysis of changes in host phenotypes across time and treatments. Evolved host populations (host coevolution, host one-sided adaptation, and host control) were exposed to the ancestral pathogen; the defined models included evolution treatment, transfer, the interaction between the two as fixed factors, and replicate nested within treatment as a random factor. The specified models provide a better fit to the data than the corresponding minimal models (*p* < 0.0001). The table shows the results for the factor effect tests, none of which yielded a significant result. The data is provided in S1 Data.
(DOCX)

S4 Table Comparison between evolved and ancestral pathogen phenotypes. Comparison between evolved (pathogen coevolution, pathogen one-sided adaptation, and pathogen control) and ancestral pathogens both exposed to ancestral hosts using an analysis of variance. Degrees of freedom (df) are given for the comparison and the error (before and after comma, respectively). Significant values after FDR adjustment are in bold. The data is provided in S1 Data.
(DOCX)

S5 Table Analysis of the changes in pathogen phenotypes across time and treatments. Evolved pathogen populations (pathogen coevolution, pathogen one-sided adaptation, and pathogen control) were exposed to the ancestral host; the defined models included evolution treatment, transfer, the interaction between the two as fixed factors, and replicate nested within treatment as a random factor. The specified models provide a better fit to the data than the corresponding minimal models (*p* < 0.0001). The table shows the results for the factor effect tests. Significant probabilities are given in bold. The data is provided in S1 Data.
(DOCX)

S6 Table Fisher exact test of differences in the number of bacterial populations able to form biofilm. The data is provided in S2 Data.
(DOCX)

S7 Table Mann-Whitney U test of differences in bacterial competition. The data is provided in S2 Data.
(DOCX)

S8 Table Statistics of the mapping of Illumina reads to the concatenated metareference. (XLSX)

S9 Table Statistics of the mapping of Illumina reads to the five different reference genomes. Table is presented as an Excel file.
(XLSX)

S10 Table Statistical analysis of the variation in strain composition across evolution treatments and time. We used an extended analysis of molecular variance (AMOVA) adonis function in R package vegan. The defined model included evolution treatment, transfer, and the interactions between the two as fixed factors and replicate nested within treatment as random factor. The specified model provided a better fit to the data than the corresponding minimal model (*p* < 0.0001). The table shows the effect tests for the fixed factors. Significant probabilities are given in bold. The data is provided in S3 Data.
(DOCX)

S11 Table Genes and primers used for PCR-based toxin screen. (DOCX)

S12 Table Number of clones with particular chromosomal background in the evolved replicate populations. (DOCX)

S13 Table Statistical analysis of the variation in toxin gene composition across evolution treatments and time. The defined nominal logistic models included evolution treatment, transfer, and the interactions between the two as fixed factors and replicate nested within treatment as random factor. The specified models provided a better fit to the data than the corresponding minimal model (*p* < 0.0001). The table shows the effect tests for the fixed factors. Significant probabilities are given in bold. The data is provided in S4 Data.
(DOCX)

S14 Table Results of the analysis of SNPs and short indels in the BT-679-dominated populations. Table is presented as an Excel file.
(XLSX)

S15 Table Results of the Pindel analysis of structural variation in the BT-679-dominated populations. Table is presented as an Excel file.
(XLSX)

S16 Table Results of the analysis of copy number variation in the BT-679-dominated populations. Table is presented as an Excel file.
(XLSX)

S17 Table Results of the analysis of horizontal gene transfer to the BT-679 genotype. Table is presented as an Excel file.
(XLSX)

S18 Table Results of the analysis of copy number variation for entire contigs in the BT-679-dominated populations. Table is presented as an Excel file.
(XLSX)

S19 Table Results for the population genetic analysis of the BT-679-dominated populations. Table is presented as an Excel file.
(XLSX)

S20 Table Summary of significant variations in the fine-scale genomic analysis of the BT-679-dominated populations. Table is presented as an Excel file.
(XLSX)

S21 Table Pairwise analysis of the deletion frequency in the mviN virulence gene, the copy number of the cry toxin-containing plasmid and killing ability. Pairwise analysis was performed twice using: (i) the Spearman rank order correlation analysis (correlation parameter *ρ*_*s*_); and (ii) weighted regression analysis (strength of relationship indicated by *R* ^*2*^), whereby plasmid copy number was weighted by its variance to reduce influence of outliers. Significant values after FDR adjustment are in bold. The data is provided in S5 Data.
(DOCX)

S22 Table Primers used during functional analysis of cry toxins. (DOCX)

S23 Table Statistical analysis of nematode survival after exposure to cry-toxin-expressing *B*. *thuringiensis*. The data is provided in S6 Data.
(DOCX)

S24 Table Statistical analysis of nematode survival after exposure to cry-toxin-expressing *E*. *coli*. The data is provided in S6 Data.
(DOCX)

## Abbreviations

AMOVA: analysis of molecular variance
FDR: false discovery rate
