# Supplementary figures and images for: Host–Pathogen Coevolution: The Selective Advantage of Bacillus thuringiensis Virulence and Its Cry Toxin Genes
Source: PLoS Biol. 2015 Jun 4;13(6):e1002169. doi: 10.1371/journal.pbio.1002169 (PMC4456383; doi:10.1371/journal.pbio.1002169)

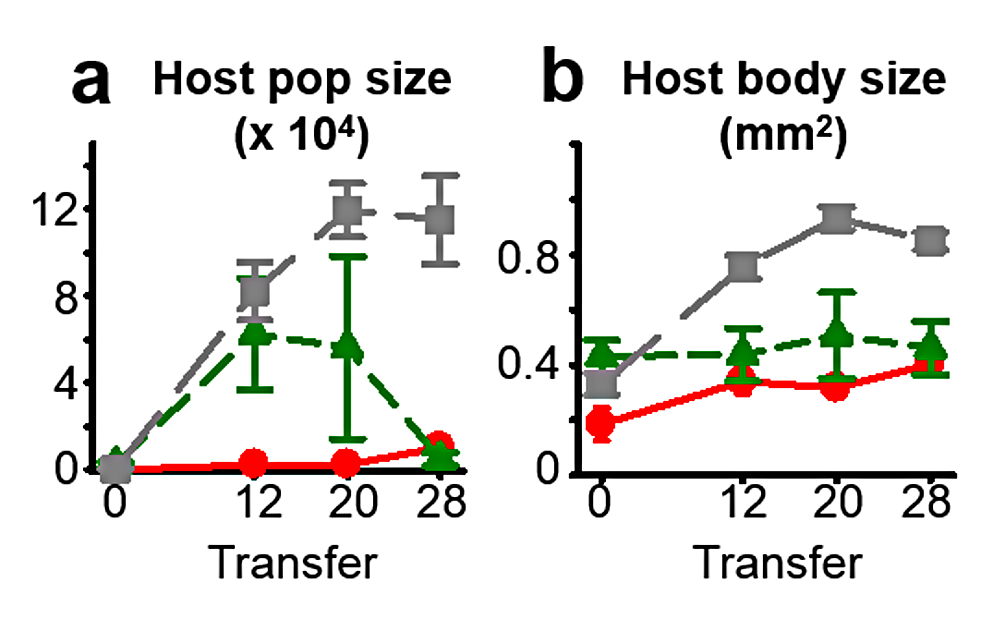

Supplement: S1 Fig — A, Variation in the pathogen's effect on host population size; and B, in the pathogen's effect on host body size. Bars show standard errors. S4 Table and S5 Table show the corresponding statistical results. The original data is provided in S1 Data. (TIF) [file pbio.1002169.s010.tif]

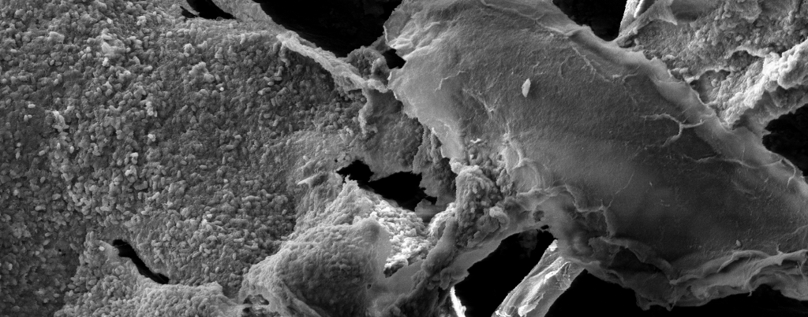

Supplement: S2 Fig — (TIF) [file pbio.1002169.s011.tif]

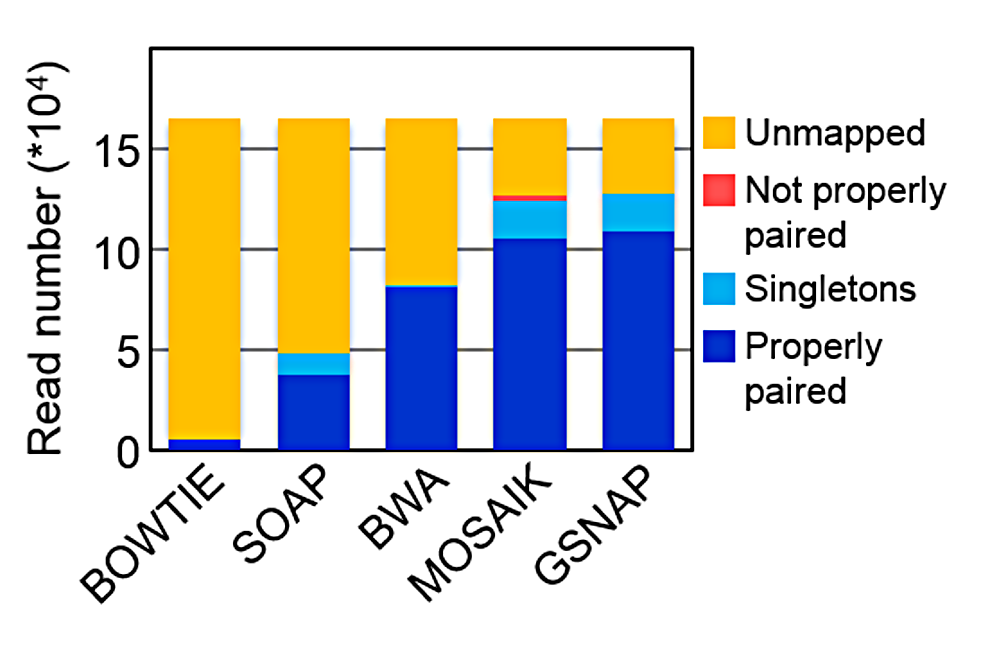

Supplement: S3 Fig — The five mapping programs are given along the x-axis. The y-axis presents the total number of reads mapped, classified in four categories following the samtools flagstat function: (i) reads unmapped (yellow bar area); (ii) not properly paired: both reads of a pair are mapped onto the reference genome but expected insert size and/or orientation is incorrect (red bar area); (iii) singletons: only one read of the pair is mapped (light blue area); and (iv) properly paired: both reads are mapped onto the reference genome with correct orientation and expected insert size (dark blue area). The data is shown in S7 Data. (TIF) [file pbio.1002169.s012.tif]

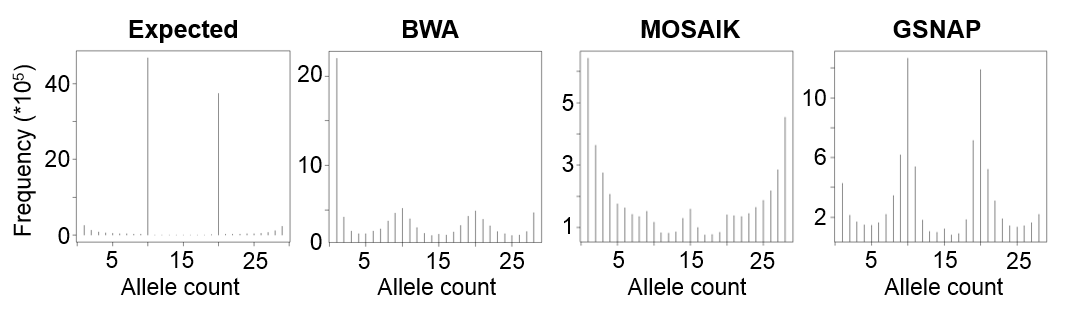

Supplement: S4 Fig — Original spectrum relative to the reference genome NC_014171.1 (expected results on far left), and the results obtained with the mapping software BWA, MOSAIK, and GSNAP. The data is shown in S8 Data. (TIF) [file pbio.1002169.s013.tif]

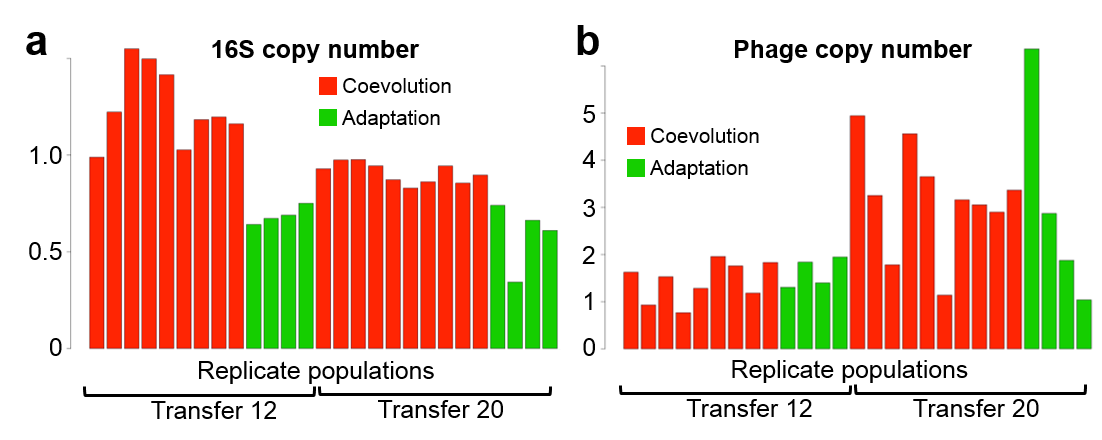

Supplement: S5 Fig — A, Significant variation among treatments for a 16S rRNA gene, horizontally transferred from the BT-50 ancestral strain to the BT-679 genotype. B, Horizontal transfer and spread of a phage from the non-nematocidal ancestral strain BT-50 in the BT-679 coevolved and one-sided adapted populations across time. The different replicate populations are given along the x-axis and DNA fragment frequency on the y-axis. Red indicates coevolution and green one-sided adaptation. The data is shown in S9 Data. (TIF) [file pbio.1002169.s014.tif]
