# Supplementary material for: Host–Pathogen Coevolution: The Selective Advantage of Bacillus thuringiensis Virulence and Its Cry Toxin Genes
Source: PLoS Biol. 2015 Jun 4;13(6):e1002169. doi: 10.1371/journal.pbio.1002169 (PMC4456383; doi:10.1371/journal.pbio.1002169)
Supplement: S1 Table — Pairwise comparison of the different exposure types. Here, the coevolved–coevolved exposures from a particular replicate population were compared with the corresponding exposures, in which the same coevolved host or pathogen replicate was confronted with the ancestral antagonist. Significant values after FDR adjustment are given in bold. The data is provided in S1 Data. (DOCX) [file pbio.1002169.s015.docx]

**S1 Table. Analysis of reciprocal coevolutionary adaptation in comparison to adaptation to the ancestral antagonist^1^**

| **Transfer** | **Comparison^2^** | ***χ^2^*** | **df** | ***P*** |
| --- | --- | --- | --- | --- |
| 12 | Co-H+Co-P vs. Co-H+Anc-P | 4.75 | 1 | **0.0293** |
|  | Co-H+Co-P vs. Anc-H+Co-P | 11.71 | 1 | **0.0006** |
| 20 | Co-H+Co-P vs. Co-H+Anc-P | 3.27 | 1 | 0.0704 |
|  | Co-H+Co-P vs. Anc-H+Co-P | 14.29 | 1 | **0.0002** |
| 28 | Co-H+Co-P vs. Co-H+Anc-P | 2.55 | 1 | 0.1102 |
|  | Co-H+Co-P vs. Anc-H+Co-P | 0.37 | 1 | 0.5418 |

^1^ General linear model analysis, based on ordinal logistic regression, with exposure type and replicate as factors. The defined model always provided a better fit than the minimal model (*P < 0.05*).

^2^ Statistical results are shown for the factor effect tests, based on the pairwise comparison of the indicated exposure types. Here, the coevolved-coevolved exposures from a particular replicate population (indicated by "Co-H+Co-P"; H for host; P for pathogen) were compared with the corresponding exposures, in which the same coevolved host or pathogen replicate was confronted with the ancestral antagonist (either "Co-H+Anc-P" or "Anc-H+Co-P"). df, degrees of freedom. *P*, probability. Significant values after FDR adjustment are given in bold. The data is shown in S1 Data.
