# Supplementary material for: Host–Pathogen Coevolution: The Selective Advantage of Bacillus thuringiensis Virulence and Its Cry Toxin Genes
Source: PLoS Biol. 2015 Jun 4;13(6):e1002169. doi: 10.1371/journal.pbio.1002169 (PMC4456383; doi:10.1371/journal.pbio.1002169)
Supplement: S3 Table — Evolved host populations (host coevolution, host one-sided adaptation, and host control) were exposed to the ancestral pathogen; the defined models included evolution treatment, transfer, the interaction between the two as fixed factors, and replicate nested within treatment as a random factor. The specified models provide a better fit to the data than the corresponding minimal models (p < 0.0001). The table shows the results for the factor effect tests, none of which yielded a significant result. The data is provided in S1 Data. (DOCX) [file pbio.1002169.s017.docx]

**S3 Table. Analysis of changes in host phenotypes across time and treatments^1^**

| **Trait and comparison** | **Factor** | **df** | ***F*** | ***P*** |
| --- | --- | --- | --- | --- |
| **Host survival** |  |  |  |  |
| Coevolution vs. Adaptation | Treatment | 1 | <0.01 | 0.9577 |
|  | Transfer | 2 | 2.23 | 0.1237 |
|  | Treat.*Trans. | 2 | 0.71 | 0.4984 |
| Coevolution vs. Control | Treatment | 1 | 1.37 | 0.2479 |
|  | Transfer | 2 | 2.46 | 0.1009 |
|  | Treat.*Trans. | 2 | 0.52 | 0.5971 |
| Adaptation vs. Control | Treatment | 1 | 3.06 | 0.0862 |
|  | Transfer | 2 | 1.58 | 0.2203 |
|  | Treat.*Trans. | 2 | 1.44 | 0.2513 |
| **Host pop. growth** |  |  |  |  |
| Coevolution vs. Adaptation | Treatment | 1 | 0.21 | 0.6531 |
|  | Transfer | 2 | 0.94 | 0.4032 |
|  | Treat.*Trans. | 2 | 0.60 | 0.5567 |
| Coevolution vs. Control | Treatment | 1 | 1.48 | 0.2323 |
|  | Transfer | 2 | 0.05 | 0.9525 |
|  | Treat.*Trans. | 2 | 1.89 | 0.1718 |
| Adaptation vs. Control | Treatment | 1 | 0.61 | 0.4407 |
|  | Transfer | 2 | 0.64 | 0.5356 |
|  | Treat.*Trans. | 2 | 1.14 | 0.3354 |
| **Host body size** |  |  |  |  |
| Coevolution vs. Adaptation | Treatment | 1 | 0.05 | 0.8162 |
|  | Transfer | 2 | 0.22 | 0.8011 |
|  | Treat.*Trans. | 2 | 2.34 | 0.1184 |
| Coevolution vs. Control | Treatment | 1 | 0.62 | 0.4353 |
|  | Transfer | 2 | 0.09 | 0.9184 |
|  | Treat.*Trans. | 2 | 0.35 | 0.7060 |
| Adaptation vs. Control | Treatment | 1 | 0.30 | 0.5893 |
|  | Transfer | 2 | 1.27 | 0.2956 |
|  | Treat.*Trans. | 2 | 0.57 | 0.5717 |
| **Host infection load** |  |  |  |  |
| Coevolution vs. Adaptation | Treatment | 1 | 0.07 | 0.7857 |
|  | Transfer | 2 | 2.91 | 0.0687 |
|  | Treat.*Trans. | 2 | 0.19 | 0.8244 |
| Coevolution vs. Control | Treatment | 1 | 0.01 | 0.9105 |
|  | Transfer | 2 | 1.99 | 0.1532 |
|  | Treat.*Trans. | 2 | 0.48 | 0.6237 |
| Adaptation vs. Control | Treatment | 1 | 0.05 | 0.8165 |
|  | Transfer | 2 | 2.25 | 0.1206 |
|  | Treat.*Trans. | 2 | 0.40 | 0.6715 |

^1^ Evolved host populations (host coevolution, host one-sided adaptation and host control) were exposed to the ancestral pathogen; the defined models included evolution treatment, transfer, the interaction between the two as fixed factors and replicate nested within treatment as a random factor. The models were assessed for all three pairwise combinations of the evolution treatments, as indicated in the left column. The specified models provide a better fit to the data than the corresponding minimal models (*P* < 0.0001). The table shows the results for the factor effect tests, none of which yielded a significant result. Significance was adjusted using FDR to take account of increased type I errors. Significant probabilities are given in bold. The data is shown in S1 Data.
