# Supplementary material for: Host–Pathogen Coevolution: The Selective Advantage of Bacillus thuringiensis Virulence and Its Cry Toxin Genes
Source: PLoS Biol. 2015 Jun 4;13(6):e1002169. doi: 10.1371/journal.pbio.1002169 (PMC4456383; doi:10.1371/journal.pbio.1002169)
Supplement: S4 Table — Comparison between evolved (pathogen coevolution, pathogen one-sided adaptation, and pathogen control) and ancestral pathogens both exposed to ancestral hosts using an analysis of variance. Degrees of freedom (df) are given for the comparison and the error (before and after comma, respectively). Significant values after FDR adjustment are in bold. The data is provided in S1 Data. (DOCX) [file pbio.1002169.s018.docx]

**S4 Table. Comparison between evolved and ancestral pathogen phenotypes^1^**

| **Trait** | **Treatment** | **Transfer^2^** | ***F*** | **df** | ***P*** |
| --- | --- | --- | --- | --- | --- |
| Killing ability | Coevolution | 12 | 0.50 | 1,16 | 0.4858 |
|  |  | 20 | 0.79 | 1,16 | 0.3869 |
|  |  | 28 | 8.53 | 1,16 | **0.0100** |
|  | Adaptation | 12 | 4.41 | 1,16 | 0.0517 |
|  |  | 20 | 0.46 | 1,11 | 0.5086 |
|  |  | 28 | 2.10 | 1,11 | 0.1748 |
|  | Control | 12 | 215.88 | 1,15 | **<0.0001** |
|  |  | 20 | 1715.68 | 1,15 | **<0.0001** |
|  |  | 28 | 1542.75 | 1,14 | **<0.0001** |
| Pathogen | Coevolution | 12 | 1.02 | 1,14 | 0.3200 |
| impact |  | 20 | 1.26 | 1,14 | 0.2700 |
| on host pop. |  | 28 | 2.94 | 1,14 | 0.1081 |
| growth | Adaptation | 12 | 3.20 | 1,14 | 0.0949 |
|  |  | 20 | 1.64 | 1,10 | 0.2287 |
|  |  | 28 | 0.79 | 1,8 | 0.3975 |
|  | Control | 12 | 21.89 | 1,14 | **0.0004** |
|  |  | 20 | 95.13 | 1,10 | **<0.0001** |
|  |  | 28 | 18.70 | 1,14 | **0.0007** |
| Pathogen | Coevolution | 12 | 4.89 | 1,14 | 0.0440 |
| impact |  | 20 | 4.19 | 1,13 | 0.0612 |
| on host body |  | 28 | 11.84 | 1,15 | **0.0036** |
| size | Adaptation | 12 | 0.00 | 1,14 | 0.9623 |
|  |  | 20 | 0.27 | 1,10 | 0.6138 |
|  |  | 28 | 0.08 | 1,10 | 0.7792 |
|  | Control | 12 | 46.68 | 1,15 | **<0.0001** |
|  |  | 20 | 111.18 | 1,11 | **<0.0001** |
|  |  | 28 | 103.81 | 1,13 | **<0.0001** |
| Pathogen | Coevolution | 12 | 2.79 | 1,14 | 0.1168 |
| infection load^3^ |  | 20 | 1.83 | 1,14 | 0.1967 |
|  |  | 28 | 3.95 | 1,15 | 0.0653 |
|  | Adaptation | 12 | 4.10 | 1,13 | 0.0638 |
|  |  | 20 | 3.40 | 1,8 | 0.1023 |
|  |  | 28 | 5.18 | 1,8 | 0.0523 |
|  | Control | 12 | 3.42 | 1,15 | 0.0842 |
|  |  | 20 | 2.26 | 1,11 | 0.1607 |
|  |  | 28 | 3.2 | 1,13 | 0.0966 |

^1^ Comparison between evolved (pathogen coevolution, pathogen one-sided adaptation and pathogen control) and ancestral pathogens both exposed to ancestral hosts using an analysis of variance. Degrees of freedom (df) are given for the comparison and the error (before and after comma, respectively). Significant values after FDR adjustment are in bold. The data is shown in S1 Data.

^2^ Time point is given as host transfer number.

^3^ Infection load is adjusted by body size.
