# Supplementary material for: Host–Pathogen Coevolution: The Selective Advantage of Bacillus thuringiensis Virulence and Its Cry Toxin Genes
Source: PLoS Biol. 2015 Jun 4;13(6):e1002169. doi: 10.1371/journal.pbio.1002169 (PMC4456383; doi:10.1371/journal.pbio.1002169)
Supplement: S5 Table — Evolved pathogen populations (pathogen coevolution, pathogen one-sided adaptation, and pathogen control) were exposed to the ancestral host; the defined models included evolution treatment, transfer, the interaction between the two as fixed factors, and replicate nested within treatment as a random factor. The specified models provide a better fit to the data than the corresponding minimal models (p < 0.0001). The table shows the results for the factor effect tests. Significant probabilities are given in bold. The data is provided in S1 Data. (DOCX) [file pbio.1002169.s019.docx]

**S5 Table. Analysis of the changes in pathogen phenotypes across time and treatments^1^**

| **Trait and comparison** | **Factor** | **df** | ***F*** | ***P*** |
| --- | --- | --- | --- | --- |
| **Killing ability** |  |  |  |  |
| Coevolution vs. Adaptation | Treatment | 1 | 6.33 | **0.0179** |
|  | Transfer | 2 | 3.41 | 0.0499 |
|  | Treat.*Trans. | 2 | 0.54 | 0.5901 |
| Coevolution vs. Control | Treatment | 1 | 100.81 | **<0.0001** |
|  | Transfer | 2 | 6.26 | **0.0046** |
|  | Treat.*Trans. | 2 | 4.39 | **0.0195** |
| Adaptation vs. Control | Treatment | 1 | 6.79 | **0.0162** |
|  | Transfer | 2 | 1.53 | 0.2368 |
|  | Treat.*Trans. | 2 | 1.15 | 0.3347 |
| **Impact on host pop. growth** |  |  |  |  |
| Coevolution vs. Adaptation | Treatment | 1 | 5.24 | 0.0333 |
|  | Transfer | 2 | 2.92 | 0.0724 |
|  | Treat.*Trans. | 2 | 2.55 | 0.0982 |
| Coevolution vs. Control | Treatment | 1 | 26.09 | **<0.0001** |
|  | Transfer | 2 | 2.23 | 0.1224 |
|  | Treat.*Trans. | 2 | 1.39 | 0.2639 |
| Adaptation vs. Control | Treatment | 1 | 0.40 | 0.5345 |
|  | Transfer | 2 | 1.06 | 0.3648 |
|  | Treat.*Trans. | 2 | 0.69 | 0.5109 |
| **Impact on host body size** |  |  |  |  |
| Coevolution vs. Adaptation | Treatment | 1 | 1.47 | 0.2376 |
|  | Transfer | 2 | 3.02 | 0.0683 |
|  | Treat.*Trans. | 2 | 2.22 | 0.1316 |
| Coevolution vs. Control | Treatment | 1 | 57.13 | **<0.0001** |
|  | Transfer | 2 | 3.36 | 0.0496 |
|  | Treat.*Trans. | 2 | 2.88 | 0.0734 |
| Adaptation vs. Control | Treatment | 1 | 13.51 | **0.0015** |
|  | Transfer | 2 | 5.63 | **0.0115** |
|  | Treat.*Trans. | 2 | 0.38 | 0.6855 |
| **Infection load** |  |  |  |  |
| Coevolution vs. Adaptation | Treatment | 1 | 9.32 | **0.0041** |
|  | Transfer | 2 | <0.01 | 0.9988 |
|  | Treat.*Trans. | 2 | 0.49 | 0.6182 |
| Coevolution vs. Control | Treatment | 1 | 0.03 | 0.8680 |
|  | Transfer | 2 | 0.96 | 0.3934 |
|  | Treat.*Trans. | 2 | 0.94 | 0.4009 |
| Adaptation vs. Control | Treatment | 1 | 11.94 | **0.0015** |
|  | Transfer | 2 | 0.11 | 0.8932 |
|  | Treat.*Trans. | 2 | 0.11 | 0.8955 |

^1^ Evolved pathogen populations (pathogen coevolution, pathogen one-sided adaptation and pathogen control) were exposed to the ancestral host; the defined models included evolution treatment, transfer, the interaction between the two as fixed factors and replicate nested within treatment as a random factor. The models were assessed for all three pairwise combinations of the evolution treatments, as indicated in the left column. The specified models provide a better fit to the data than the corresponding minimal models (*P* < 0.0001). The table shows the results for the factor effect tests, none of which yielded a significant result. Significance was adjusted using FDR to take account of increased type I errors. Significant probabilities are given in bold. The data is shown in S1 Data.
