# Supplementary material for: Host–Pathogen Coevolution: The Selective Advantage of Bacillus thuringiensis Virulence and Its Cry Toxin Genes
Source: PLoS Biol. 2015 Jun 4;13(6):e1002169. doi: 10.1371/journal.pbio.1002169 (PMC4456383; doi:10.1371/journal.pbio.1002169)
Supplement: S6 Table — The data is provided in S2 Data. (DOCX) [file pbio.1002169.s020.docx]

**S6 Table. Fisher exact test of differences in the number of bacterial populations able to form biofilm^1^**

| **Comparison** | **Transfer** | df | N^2^ | *P* |
| --- | --- | --- | --- | --- |
| Coevolution vs. Adaptation | 12 | 1 | 10,9 | **0.0031** |
| Coevolution vs. Control | 12 | 1 | 10,10 | **<0.0001** |
| Control vs. Adaptation | 12 | 1 | 10,9 | 0.0867 |
| Coevolution vs. Adaptation | 20 | 1 | 10,7 | **0.0034** |
| Coevolution vs. Control | 20 | 1 | 10,8 | **<0.0001** |
| Control vs. Adaptation | 20 | 1 | 8,7 | 0.2000 |
| Coevolution vs. Adaptation | 28 | 1 | 10,4 | 0.2857 |
| Coevolution vs. Control | 28 | 1 | 10,8 | **<0.0001** |
| Control vs. Adaptation | 28 | 1 | 8,4 | **0.0182** |

^1^ Time point is given as host transfer number. Significant values after FDR adjustment are given in bold. The data is shown in S2 Data.

^2^ Sample sizes for first and second factor of the comparison, respectively.
