# Supplementary material for: Host–Pathogen Coevolution: The Selective Advantage of Bacillus thuringiensis Virulence and Its Cry Toxin Genes
Source: PLoS Biol. 2015 Jun 4;13(6):e1002169. doi: 10.1371/journal.pbio.1002169 (PMC4456383; doi:10.1371/journal.pbio.1002169)
Supplement: S7 Table — The data is provided in S2 Data. (DOCX) [file pbio.1002169.s021.docx]

**S7 Table. Mann-Whitney U test of differences in bacterial competition**

| **Medium^1^** | **Comparison^2^** | ***U*** | **N^3^** | ***P*^4^** |
| --- | --- | --- | --- | --- |
| NGM | B/B vs. NB/NB | 1.06 | 44, 45 | 0.2870 |
|  | B/B vs. NB/B | 5.34 | 44, 59 | **<0.0001** |
|  | NB/B vs. NB/NB | 4.86 | 59, 45 | **<0.0001** |
| PF-NGM | B/B vs. NB/NB | 1.02 | 14, 14 | 0.3071 |
|  | B/B vs. NB/B | -2.28 | 14, 23 | **0.0221** |
|  | NB/B vs. NB/NB | -1.48 | 23, 14 | 0.1333 |

^1^ NGM, nutrient-rich Nematode Growth Medium; PFM, nutrient-poor Peptone-Free Medium

^2^ B, biofilm-forming bacterial clone; NB, non-biofilm-forming clone

^3^ Sample sizes for first and second factor of the comparison, respectively.

^4^ Significant probabilities after FDR correction are given in bold. The data is shown in S2 Data.
