# Supplementary material for: Host–Pathogen Coevolution: The Selective Advantage of Bacillus thuringiensis Virulence and Its Cry Toxin Genes
Source: PLoS Biol. 2015 Jun 4;13(6):e1002169. doi: 10.1371/journal.pbio.1002169 (PMC4456383; doi:10.1371/journal.pbio.1002169)
Supplement: S10 Table — We used an extended analysis of molecular variance (AMOVA) adonis function in R package vegan. The defined model included evolution treatment, transfer, and the interactions between the two as fixed factors and replicate nested within treatment as random factor. The specified model provided a better fit to the data than the corresponding minimal model (p < 0.0001). The table shows the effect tests for the fixed factors. Significant probabilities are given in bold. The data is provided in S3 Data. (DOCX) [file pbio.1002169.s024.docx]

**S10 Table. Statistical analysis of the variation in strain composition across evolution treatments and time^1^.**

| **Comparison** | **Factor** | **Df** | ***F*** | ***P*** |
| --- | --- | --- | --- | --- |
| Coevolution vs. Adaptation | Treatment | 2 | 15.5 | **0.001** |
|  | Transfer | 1 | 6.0 | **0.004** |
|  | Treatment * Transfer | 2 | 1.4 | 0.240 |
| Coevolution vs. Control | Treatment | 4 | 19.8 | **0.001** |
|  | Transfer | 1 | 6.0 | **0.010** |
|  | Treatment * Transfer | 3 | 1.7 | 0.155 |
| Adaptation vs. Control | Treatment | 2 | 51.3 | **0.001** |
|  | Transfer | 1 | 1.0 | 0.332 |
|  | Treatment * Transfer | 2 | 0.8 | 0.459 |

^1^ We used an extended AMOVA adonis function in R package vegan. The defined model included evolution treatment, transfer, and the interactions between the two as fixed factors and replicate nested within treatment as random factor. The specified model provided a better fit to the data than the corresponding minimal model (*P* < 0.0001). The table shows the effect tests for the fixed factors. Significant probabilities after FDR adjustment are given in bold. The data is shown in S3 Data.
