# Supplementary material for: Host–Pathogen Coevolution: The Selective Advantage of Bacillus thuringiensis Virulence and Its Cry Toxin Genes
Source: PLoS Biol. 2015 Jun 4;13(6):e1002169. doi: 10.1371/journal.pbio.1002169 (PMC4456383; doi:10.1371/journal.pbio.1002169)
Supplement: S11 Table — (DOCX) [file pbio.1002169.s025.docx]

S11 Table. Genes and primers used for PCR-based toxin screen.

| **Gene** | **Strain** | **Forward primer (5'-3')** | **Reverse primer (5'-3')** |
| --- | --- | --- | --- |
| *cry13Aa1* | BT-246 | AATGTGCTGGGACAATCAGG | TTGGGAATTTTCTGGAACACC |
| *cry6Ba1* | BT-247 | CTGTTCAAGTACAACTAGCAC | GGCTATCTCTTTCCATTGACC |
| *cry14Aa1* | BT-679 | CTAATAATGCGCGACCTACTG | GTACCAGCTATTGCACAACC |
| *cry21Aa2* | BT-679 | CAACACCTTCAAATCGCATGG | CATAAGTCCTGGTTGTTCTCC |
| *cry35Aa4* | BT-679 | CCAGAAGTAGGAGGAGGTACA | TTCATACCGAATGGTTTGTGAG |
| *codY* | all | TGAACACCAGCTTCAAGCAAT | GTTATTACAGAGCGCAGCAGG |
