# Supplementary material for: Host–Pathogen Coevolution: The Selective Advantage of Bacillus thuringiensis Virulence and Its Cry Toxin Genes
Source: PLoS Biol. 2015 Jun 4;13(6):e1002169. doi: 10.1371/journal.pbio.1002169 (PMC4456383; doi:10.1371/journal.pbio.1002169)
Supplement: S12 Table — (DOCX) [file pbio.1002169.s026.docx]

**S12 Table. Number of clones with particular chromosomal background in the evolved replicate populations.**

| **Treatment** | **Transfer** | **Repl.** | **BT-246** | **BT-22** | **BT-50** | **BT-679** | **Total** |
| --- | --- | --- | --- | --- | --- | --- | --- |
| Coevolution | 12 | 1 | 0 | 1 | 1 | 18 | 20 |
|  |  | 2 | 0 | 0 | 0 | 20 | 20 |
|  |  | 3 | 0 | 1 | 0 | 19 | 20 |
|  |  | 4 | 0 | 0 | 0 | 20 | 20 |
|  |  | 5 | 0 | 1 | 0 | 19 | 20 |
|  |  | 6 | 0 | 0 | 0 | 20 | 20 |
|  |  | 7 | 0 | 0 | 0 | 20 | 20 |
|  |  | 8 | 0 | 0 | 0 | 20 | 20 |
|  |  | 9 | 0 | 0 | 0 | 20 | 20 |
|  |  | 10 | 0 | 0 | 0 | 20 | 20 |
| Coevolution | 20 | 1 | 0 | 0 | 0 | 20 | 20 |
|  |  | 2 | 0 | 0 | 0 | 20 | 20 |
|  |  | 3 | 0 | 0 | 0 | 20 | 20 |
|  |  | 4 | 0 | 0 | 0 | 20 | 20 |
|  |  | 5 | 0 | 0 | 0 | 20 | 20 |
|  |  | 6 | 0 | 0 | 0 | 20 | 20 |
|  |  | 7 | 1 | 0 | 0 | 19 | 20 |
|  |  | 8 | 0 | 3 | 0 | 17 | 20 |
|  |  | 9 | 0 | 0 | 0 | 20 | 20 |
|  |  | 10 | 0 | 0 | 0 | 20 | 20 |
| Control | 12 | 1 | 0 | 19 | 0 | 1 | 20 |
|  |  | 2 | 0 | 20 | 0 | 0 | 20 |
|  |  | 3 | 0 | 19 | 1 | 0 | 20 |
|  |  | 4 | 0 | 15 | 0 | 5 | 20 |
|  |  | 5 | 0 | 2 | 18 | 0 | 20 |
|  |  | 6 | 0 | 9 | 11 | 0 | 20 |
|  |  | 7 | 0 | 12 | 8 | 0 | 20 |
|  |  | 8 | 0 | 17 | 3 | 0 | 20 |
|  |  | 9 | 0 | 11 | 6 | 3 | 20 |
|  |  | 10 | 0 | 17 | 3 | 0 | 20 |
| Control | 20 | 1 | 0 | 20 | 0 | 0 | 20 |
|  |  | 2 | 0 | 20 | 0 | 0 | 20 |
|  |  | 3 | 0 | 20 | 0 | 0 | 20 |
|  |  | 4 | 0 | 20 | 0 | 0 | 20 |
|  |  | 5 | 0 | 20 | 0 | 0 | 20 |
|  |  | 6 | 0 | 20 | 0 | 0 | 20 |
|  |  | 7 | 0 | 20 | 0 | 0 | 20 |
|  |  | 9 | 0 | 20 | 0 | 0 | 20 |
|  |  | 10 | 0 | 20 | 0 | 0 | 20 |
| Adaptation | 12 | 1 | 0 | 20 | 0 | 0 | 20 |
|  |  | 2 | 0 | 0 | 0 | 20 | 20 |
|  |  | 3 | 0 | 0 | 0 | 20 | 20 |
|  |  | 4 | 0 | 0 | 0 | 20 | 20 |
|  |  | 5 | 0 | 20 | 0 | 0 | 20 |
|  |  | 6 | 0 | 0 | 0 | 20 | 20 |
|  |  | 7 | 0 | 2 | 18 | 0 | 20 |
|  |  | 8 | 0 | 1 | 19 | 0 | 20 |
|  |  | 9 | 0 | 0 | 12 | 8 | 20 |
|  |  | 10 | 0 | 0 | 20 | 0 | 20 |
| Adaptation | 20 | 2 | 0 | 20 | 0 | 0 | 20 |
|  |  | 3 | 0 | 0 | 1 | 19 | 20 |
|  |  | 4 | 0 | 1 | 0 | 19 | 20 |
|  |  | 6 | 0 | 0 | 0 | 20 | 20 |
|  |  | 9 | 0 | 0 | 0 | 20 | 20 |
|  |  | 10 | 0 | 0 | 20 | 0 | 20 |
