# Supplementary material for: Host–Pathogen Coevolution: The Selective Advantage of Bacillus thuringiensis Virulence and Its Cry Toxin Genes
Source: PLoS Biol. 2015 Jun 4;13(6):e1002169. doi: 10.1371/journal.pbio.1002169 (PMC4456383; doi:10.1371/journal.pbio.1002169)
Supplement: S13 Table — The defined nominal logistic models included evolution treatment, transfer, and the interactions between the two as fixed factors and replicate nested within treatment as random factor. The specified models provided a better fit to the data than the corresponding minimal model (p < 0.0001). The table shows the effect tests for the fixed factors. Significant probabilities are given in bold. The data is provided in S4 Data. (DOCX) [file pbio.1002169.s027.docx]

**S13 Table. Statistical analysis of the variation in toxin gene composition across evolution treatments and time^1^.**

| **Comparison** | **Factor** | **df** | ***χ^2^*** | ***P*** |
| --- | --- | --- | --- | --- |
| Coevolution vs. Adaptation | Treatment | 5 | < 0.01 | >0.99 |
|  | Transfer | 5 | 10.9 | 0.0544 |
|  | Treatment * Transfer | 5 | 48.3 | **< 0.0001** |
| Coevolution vs. Control | Treatment | 5 | 96.3 | **< 0.0001** |
|  | Transfer | 5 | 7.1 | 0.2129 |
|  | Treatment * Transfer | 5 | 54.5 | **< 0.0001** |
| Adaptation vs. Control | Treatment | 4 | < 0.01 | > 0.99 |
|  | Transfer | 4 | 69.6 | **< 0.0001** |
|  | Treatment * Transfer | 4 | 2.55 | 0.6358 |

^1^ The defined nominal logistic models included evolution treatment, transfer, and the interactions between the two as fixed factors and replicate nested within treatment as random factor. The specified models provided a better fit to the data than the corresponding minimal model (*P* < 0.0001). The table shows the effect tests for the fixed factors. Significant probabilities after FDR correction are given in bold. The data is shown in S4 Data.
