# Supplementary material for: Host–Pathogen Coevolution: The Selective Advantage of Bacillus thuringiensis Virulence and Its Cry Toxin Genes
Source: PLoS Biol. 2015 Jun 4;13(6):e1002169. doi: 10.1371/journal.pbio.1002169 (PMC4456383; doi:10.1371/journal.pbio.1002169)
Supplement: S21 Table — Pairwise analysis was performed twice using: (i) the Spearman rank order correlation analysis (correlation parameter ρ s); and (ii) weighted regression analysis (strength of relationship indicated by R 2), whereby plasmid copy number was weighted by its variance to reduce influence of outliers. Significant values after FDR adjustment are in bold. The data is provided in S5 Data. (DOCX) [file pbio.1002169.s035.docx]

**S21 Table. Pairwise analysis of the deletion frequency in the mviN domain gene, the copy number of the cry toxin-containing plasmid and killing ability^1^**

| **Comparison** | ***ρ_s_* or *R^2^*** | ***P*** |
| --- | --- | --- |
| **Spearman rank correlation** |  |  |
| Deletion frequency vs. Killing ability | -0.48 | **0.01815** |
| Deletion frequency vs. Plasmid copy number | -0.53 | **0.0042** |
| Plasmid copy number vs. Killing ability | 0.02 | 0.9405 |
| **Weighted regression analysis** |  |  |
| Deletion frequency vs. Killing ability | 0.45 | **< 0.0001** |
| Deletion frequency vs. Plasmid copy number | 0.79 | **< 0.0001** |
| Plasmid copy number vs. Killing ability | 0.74 | **< 0.0001** |

^1^ Pairwise analysis was performed twice using: (i) the Spearman rank correlation analysis (correlation parameter *ρ_s_*); and (ii) weighted regression analysis (strength of relationship indicated by *R^2^*), whereby plasmid copy number was weighted by the inverse of its variance to reduce the influence of values with estimation inaccuracy. Significant values after FDR adjustment are in bold. The data is shown in S5 Data.
