# Supplementary material for: Host–Pathogen Coevolution: The Selective Advantage of Bacillus thuringiensis Virulence and Its Cry Toxin Genes
Source: PLoS Biol. 2015 Jun 4;13(6):e1002169. doi: 10.1371/journal.pbio.1002169 (PMC4456383; doi:10.1371/journal.pbio.1002169)
Supplement: S22 Table — (DOCX) [file pbio.1002169.s036.docx]

**S22 Table.** Primers used during functional analysis of cry toxins

| **Primer** | **Sequence (5'-3')** |
| --- | --- |
| MCS_f | GATGACGGTGAAAACCTCTG |
| MCS_r | GCCTTTGAGTGAGCTGATACC |
| 21clo-Fc | GCGGTCGACgaaaggaggtttattaaaATGACAAATCCAACTATACTATATC |
| 21clo-R | GCGGGGCATGCGATTAAGAAACGAGATGAATAC |
| 14Aclo-Fc | GCGGTCGACgaaaggaggtttattaaaATGGATTGTAATTTACAATCAC |
| 14Aclo-R | GCGGGGCATGCTGTATGGTGAGATTTACAAG |
