# Supplementary material for: Host–Pathogen Coevolution: The Selective Advantage of Bacillus thuringiensis Virulence and Its Cry Toxin Genes
Source: PLoS Biol. 2015 Jun 4;13(6):e1002169. doi: 10.1371/journal.pbio.1002169 (PMC4456383; doi:10.1371/journal.pbio.1002169)
Supplement: S23 Table — The data is provided in S6 Data. (DOCX) [file pbio.1002169.s037.docx]

**S23 Table.** Statistical analysis of nematode survival after exposure to cry-toxin-expressing *B. thuringiensis*^1^

| **Reference** | **Comparison** | ***Z*** | ***P*** |
| --- | --- | --- | --- |
| BT-679_Cry- | BT-679_Cry+ | 3.44 | **0.0023** |
|  | BT-679_Cry-_+14 | 3.05 | **0.0092** |
|  | BT-679_Cry-_+21 | 1.62 | 0.4190 |
|  | BT-679_Cry-_0 | 0.04 | >0.99 |
| BT-679_Cry+ | BT-679_Cry-_+14 | 0.35 | >0.99 |
|  | BT-679_Cry-_+21 | 1.77 | 0.3045 |
|  | BT-679_Cry-_0 | 3.35 | **0.0032** |

^1^ Non-parametric comparisons with control using Dunn method for joint ranking, as implemented in JMP 9.0.2 (SAS Institute Inc.). Significant probabilities are given in bold. Strain abbreviations: BT-679_Cry-, BT-679 without plasmid which contains the toxin genes *cry14Aa1* and *cry21Aa2*; BT-679_Cry+, BT-679 wildtype with the toxin-containing plasmids; BT-679_Cry-_+14, BT-679_Cry- with a *cry14Aa1*-expressing vector; BT-679_Cry-_+21, BT-679_Cry- with a *cry21Aa2*-expressing vector; BT-679_Cry-_+0, BT-679_Cry- with a *rfp*-expressing vector as a negative control. The data is shown in S6 Data.
